# Supplementary material for: Receiver operating characteristic curve analysis of clinical signs for screening of convergence insufficiency in young adults
Source: PLoS One. 2020 Jan 24;15(1):e0228313. doi: 10.1371/journal.pone.0228313 (PMC6980576; doi:10.1371/journal.pone.0228313)
Supplement: Attachment — Submitted filename: PLOS One_Reviewer PONE-D-19-17984.pdf [file pone.0228313.s002.pdf]

REVIEWER'S COMMENTS

- **SUMMARY:**

The article provides an analysis of differentiating which clinical tests that are best to be performed to diagnose a common binocular vision anomaly, i.e. the convergence insufficiency (CI). The complexity of the anomaly makes practitioners confused and sometimes missed out in detecting and diagnosing CI. As such, this article highlights the important clinical tests and criteria that could help practitioner by performing the Receiver operating characteristic (ROC) curve analysis. While the ROC analysis were well performed, there are a few issues that needs to be addressed as stated below.

- **FEEDBACK:**

1. **Materials & Methods Section**
   - **Line 99**
     - Please also clarify if participants recruited have not had any vision therapy or eye exercise treatment prior to the enrolment as research participant.
   - **Line 112**
     - The authors should detailed the NPC measurement whether it was done in a free space using accommodative fixation target or using the RAF ruler.
     - **MAJOR**: Target used for the test (20/50) was too large for the 20/20 research participants group. Using this target will not exert the best binocular vergence ability of the participants and would affect the whole test outcomes. Please clarify.
   - The authors should specify if the procedures were performed by a sole researcher or a group of researchers. Please also state if the procedures were done in the same order for each participant as some binocular vision assessments are invasive, i.e. will likely introduce fatigue to participants if it is not controlled.

2. **Results Section**
   - **Line 207**
     - Instead of writing the refractive errors and classify them for 'each eye', I would suggest that the authors change the way of writing this according to individual as convergence insufficiency is a binocular anomaly and not a monocular phenomenon.
   - **Line 219**
     - It would be beneficial if the authors add in the normative data (e.g.: Morgan's Normative Data on Binocular Vision) in Table 2 for the readers to understand the distribution of the data against the normative data used clinically.
   - **Line 269**
     - The NPC cut-off written in the paragraph was >7.3cm, different than what is stated in Table 5. Please double-check.

- The sensitivity and specificity for ROC analysis are usually expressed in percentage with 95% confidence interval. The authors may want to revise how they written this to make it more readable for other researchers.

3. Discussion Section

- Although the participants' groupings and parameters are complicated but the authors managed to write and explain in a good flow. I hope to see a more analytical explanation when discussing the outputs and its relationship to what have been the current knowledge in diagnosing convergence insufficiency. Some comments I have stated above may or may not change the outputs and interpretation of the results.

4. Grammatical errors

- **Line 95 & 97**

> **Subjects**
>
> The participants were 184 university students (age = 18-28 years; mean age = 22.23 ± 2.26 years) who underwent an eye examination due to ocular discomfort. This study was approved by the Kangwon National University Institutional Review Board (KWNUIRB-2019-02-001-002) and adhered to the tenets of the Declaration of Helsinki. We excluded patients with ocular diseases such as glaucoma, cataract, and retinal disease and those with a history of prior surgery, which was determined by history-taking.

- **Line 107 & 108**

> **General procedure**
>
> Before refraction assessments, all participants underwent a case evaluation to obtain information about the ocular discomfort, followed by an ocular motility and a cover-uncover test to rule out strabismus. Refraction and binocular vision tests were performed using a phoropter (VT-SE; Topcon Corporation, Tokyo, Japan) and visual charts (ACP-8; Topcon Corporation, Tokyo, Japan) at distance (6 m) or *(this should be written as* ***'and'****)* near (40 cm).

- **Line 157**

BAF was measured by using a ±2.00 D binocular flipper lens to evaluate the ability of the accommodative response at near [21]. If the subject reported clear vision for a 20/30 letter target when +2.00 D was placed in both eyes, the lens was flipped to place -2.00 D in both eyes. When the subject again reported clear vision, this indicated one cycle. Measured values were in terms of the number of cycles per

- **Line 311**

accommodative excess (AE) [20]. CI combined with accommodative functions can also exist because the vergence and accommodative systems are linked [32]. Therefore, CI should be distinguished from these conditions such as normal vision, diverse vergence and accommodative disorders, and other binocular anomalies. Our study factored these conditions to investigate the discriminative ability of each test for CI screening by performing ROC curve analysis under these conditions.
