# Supplementary material for: Receiver operating characteristic curve analysis of clinical signs for screening of convergence insufficiency in young adults
Source: PLoS One. 2020 Jan 24;15(1):e0228313. doi: 10.1371/journal.pone.0228313 (PMC6980576; doi:10.1371/journal.pone.0228313)
Supplement: Attachment — Submitted filename: PONE-D-19-17984_R1_(2nd Review).pdf [file pone.0228313.s004.pdf]

PLOS ONE

Receiver operating characteristic curve analysis of clinical signs for screening of convergence insufficiency in young adults

--Manuscript Draft--

**Manuscript Number:**	PONE-D-19-17984R1
**Article Type:**	Research Article
**Full Title:**	Receiver operating characteristic curve analysis of clinical signs for screening of convergence insufficiency in young adults
**Short Title:**	Receiver operating characteristic curve analysis for screening of convergence insufficiency
**Corresponding Author:**	Dong-Sik Yu, Ph.D.<br>Kangwon National University - Samcheok Campus<br>Samcheok, Gangwon-do KOREA, REPUBLIC OF
**Keywords:**	convergence insufficiency; BINOCULAR VISION; Sheard's criterion; Percival's criterion; near point of convergence; receiver operating characteristic curve
**Abstract:**	Convergence insufficiency (CI) is a common binocular vision disorder that is associated with various signs and symptoms in near work. However, CI screening is performed less frequently in adults than in children. We aimed to evaluate the ability of screening tests to discriminate CI from other binocular vision anomalies and normal binocular vision in young adults. One hundred eighty-four university students (age, 18-28 years) who underwent an eye examination due to ocular discomfort were included. Near point of convergence (NPC), phoria, accommodative amplitude, fusional vergence, the ratio of accommodative convergence to accommodation, relative accommodation, binocular accommodative facility, vergence facility, and the values corresponding to Sheard's and Percival's criteria were evaluated. Receiver operating characteristic (ROC) curve analysis for each test was also performed. The prevalence of CI ranged from 10.3% to 21.2%, depending on the signs and the presence of CI associated with accommodative disorders. Assessments based on NPC, Sheard's criterion, and Percival's criterion showed high discriminative ability, with the ability being higher between the CI and normal binocular vision groups than between the CI and non-CI groups. Sheard's criterion showed the highest diagnostic performance in discriminating CI with 3 signs from the non-CI group. The cut-off values were 7.3 cm for NPC, -0.23 to 1.00 for Sheard's criterion, and -4.00 to -2.33 for Percival's criterion. Our results suggest that the use of Sheard's criterion with NPC shows high performance for screening of CI.
**Order of Authors:**	Byeong-Yeon Moon
	Sang-Yeob Kim
	Dong-Sik Yu, Ph.D.
**Response to Reviewers:**	Dear reviewers:<br>Thank you for reviewing our manuscript PONE-D-19-17984, titled "Receiver operating characteristic curve analysis of clinical signs for screening of convergence insufficiency in young adults" submitted for publication in PLOS ONE.<br><br>We have revised the manuscript based the reviewers' comments. Enclosed, please find the revised manuscript with the changes highlighted in colored text.<br><br>All the authors express their appreciation for your kind consideration of our manuscript.<br>Sincerely,
**Additional Information:**	
**Question**	**Response**
**Financial Disclosure**<br><br>Enter a financial disclosure statement that	The author(s) received no specific funding for this work.

Powered by Editorial Manager® and ProduXion Manager® from Aries Systems Corporation

describes the sources of funding for the work included in this submission. Review the submission guidelines for detailed requirements. View published research articles from *PLOS ONE* for specific examples.<br><br>This statement is required for submission and **will appear in the published article** if the submission is accepted. Please make sure it is accurate.<br><br>**Unfunded studies**<br>Enter: *The author(s) received no specific funding for this work.*<br><br>**Funded studies**<br>Enter a statement with the following details:<br>• Initials of the authors who received each award<br>• Grant numbers awarded to each author<br>• The full name of each funder<br>• URL of each funder website<br>• Did the sponsors or funders play any role in the study design, data collection and analysis, decision to publish, or preparation of the manuscript?<br>• **NO** - Include this sentence at the end of your statement: *The funders had no role in study design, data collection and analysis, decision to publish, or preparation of the manuscript.*<br>• **YES** - Specify the role(s) played.<br><br>* typeset	
**Competing Interests**<br><br>Use the instructions below to enter a competing interest statement for this submission. On behalf of all authors, disclose any competing interests that could be perceived to bias this work—acknowledging all financial support and any other relevant financial or non-financial competing interests.<br><br>This statement **will appear in the published article** if the submission is accepted. Please make sure it is accurate. View published research articles from *PLOS ONE* for specific examples.	The authors have declared that no competing interests exist.

**NO authors have competing interests**<br><br>Enter: *The authors have declared that no competing interests exist.*<br><br>**Authors with competing interests**<br><br>Enter competing interest details beginning with this statement:<br><br>*I have read the journal's policy and the authors of this manuscript have the following competing interests: [insert competing interests here]*<br><br>* typeset	
**Ethics Statement**<br><br>Enter an ethics statement for this submission. This statement is required if the study involved:<br><br>• Human participants<br>• Human specimens or tissue<br>• Vertebrate animals or cephalopods<br>• Vertebrate embryos or tissues<br>• Field research<br><br>Write "N/A" if the submission does not require an ethics statement.<br><br>General guidance is provided below. Consult the submission guidelines for detailed instructions. **Make sure that all information entered here is included in the Methods section of the manuscript.**	This study was approved by the Kangwon National University Institutional Review Board (KWNUIRB-2019-02-001- 002), with waiver of the informed consent for the retrospective collection of clinical data, and adhered to the tenets of the Declaration of Helsinki.

Powered by Editorial Manager® and ProduXion Manager® from Aries Systems Corporation

**Format for specific study types**<br><br>**Human Subject Research (involving human participants and/or tissue)**<br>• Give the name of the institutional review board or ethics committee that approved the study<br>• Include the approval number and/or a statement indicating approval of this research<br>• Indicate the form of consent obtained (written/oral) or the reason that consent was not obtained (e.g. the data were analyzed anonymously)<br><br>**Animal Research (involving vertebrate animals, embryos or tissues)**<br>• Provide the name of the Institutional Animal Care and Use Committee (IACUC) or other relevant ethics board that reviewed the study protocol, and indicate whether they approved this research or granted a formal waiver of ethical approval<br>• Include an approval number if one was obtained<br>• If the study involved *non-human primates*, add *additional details* about animal welfare and steps taken to ameliorate suffering<br>• If anesthesia, euthanasia, or any kind of animal sacrifice is part of the study, include briefly which substances and/or methods were applied<br><br>**Field Research**<br><br>Include the following details if this study involves the collection of plant, animal, or other materials from a natural setting:<br>• Field permit number<br>• Name of the institution or relevant body that granted permission	
**Data Availability**<br><br>Authors are required to make all data underlying the findings described fully available, without restriction, and from the time of publication. PLOS allows rare exceptions to address legal and ethical concerns. See the PLOS Data Policy and FAQ for detailed information.	Yes - all data are fully available without restriction

Powered by Editorial Manager® and ProduXion Manager® from Aries Systems Corporation

A Data Availability Statement describing where the data can be found is required at submission. Your answers to this question constitute the Data Availability Statement and **will be published in the article**, if accepted.<br><br>**Important:** Stating 'data available on request from the author' is not sufficient. If your data are only available upon request, select 'No' for the first question and explain your exceptional situation in the text box.<br><br>Do the authors confirm that all data underlying the findings described in their manuscript are fully available without restriction?	
**Describe where the data may be found in full sentences. If you are copying our sample text, replace any instances of XXX with the appropriate details.**<br><br>• If the data are **held or will be held in a public repository**, include URLs, accession numbers or DOIs. If this information will only be available after acceptance, indicate this by ticking the box below. For example: *All XXX files are available from the XXX database (accession number(s) XXX, XXX.*).<br>• If the data are all contained **within the manuscript and/or Supporting Information files**, enter the following: *All relevant data are within the manuscript and its Supporting Information files.*<br>• If neither of these applies but you are able to provide **details of access elsewhere**, with or without limitations, please do so. For example:<br><br>*Data cannot be shared publicly because of [XXX]. Data are available from the XXX Institutional Data Access / Ethics Committee (contact via XXX) for researchers who meet the criteria for access to confidential data.*<br><br>*The data underlying the results presented in the study are available from (include the name of the third party*	All relevant data are within the manuscript and its Supporting Information files.

Powered by Editorial Manager® and ProduXion Manager® from Aries Systems Corporation

and contact information or URL).<br>• This text is appropriate if the data are owned by a third party and authors do not have permission to share the data.<br><br>* typeset	
Additional data availability information:	

Powered by Editorial Manager® and ProduXion Manager® from Aries Systems Corporation

Manuscript

Click here to access/download;Manuscript;Revised_Manuscript.docx

Receiver operating characteristic curve analysis of clinical signs for screening of convergence insufficiency in young adults

Byeong-Yeon Moon[1], Sang-Yeob Kim[1], Dong-Sik Yu[1*]

[1]Department of Optometry, Kangwon National University, Samcheok, Korea

* Corresponding author

E-mail: yds@kangwon.ac.kr

Abstract

Convergence insufficiency (CI) is a dysfunction of binocular vision that is associated with various signs and symptoms in near work. However, CI screening is performed less frequently in adults than in children. We aimed to evaluate the ability of screening tests to discriminate CI from other binocular vision anomalies and normal binocular vision in young adults. One hundred eighty-four university students (age, 18-28 years) who underwent an eye examination due to ocular discomfort were included. Near point of convergence (NPC), phoria, accommodative amplitude, fusional vergence, the ratio of accommodative convergence to accommodation, relative accommodation, binocular accommodative facility, vergence facility, and the values corresponding to Sheard's and Percival's criteria were evaluated. Receiver operating characteristic (ROC) curve analysis for each test was also performed. The prevalence of CI ranged from 10.3% to 21.2%, depending on the signs and the presence of CI associated with accommodative disorders. Assessments based on NPC, Sheard's criterion, and Percival's criterion showed high discriminative ability, with the ability being higher between the CI and normal binocular vision groups than between the CI and non-CI groups. Sheard's criterion showed the highest diagnostic performance in discriminating CI with three signs from the non-CI group. The cut-off values were 7.2 cm for NPC, -0.23 to 1.00 for Sheard's criterion, and -4.00 to -2.33 for Percival's criterion. Our results suggest that the use of Sheard's criterion with NPC shows high performance for screening of CI.

Introduction

Non-strabismic binocular vision anomalies consist mostly of accommodative and vergence disorders. Vergence disorders are classified into convergence insufficiency (CI), convergence excess, divergence insufficiency, divergence excess, basic esophoria, basic exophoria, and fusional vergence dysfunctions based on phoria and the accommodative convergence to accommodation ratio (AC/A) [1, 2]. CI is a dysfunction of binocular vision that is associated with various symptoms, including blurred vision, diplopia, and discomfort while performing near work [3]. The signs of CI include exophoria that is greater at near than at distance, reduced near point of convergence (NPC), reduced positive fusional vergence (PFV), low AC/A, and deficiencies in negative relative accommodation (NRA) [1]. However, cases with CI can be simple or complex aspects because not all patients with CI have all these symptoms and signs [1, 4]. The reported prevalence of CI varies widely from 1.0% to 33% [1, 5-7]. These wide variations can be attributed to the differences in patient age, sample populations, and diagnostic criteria used in the previous studies [8]. Therefore, it is important to evaluate the ability of clinical diagnostic tests to discriminate between CI and other disorders, since the clinical criteria used for screening CI have been different across several studies.

Receiver operating characteristic (ROC) curve analysis is useful for evaluating the quality or performance of diagnostic tests. In a very recent study [9], ROC curve analysis was used to assess the performance of PFV, NPC, AC/A, accommodative amplitude (AA), binocular accommodative facility (BAF), ratio of PFV over the phoria (PFV/phoria), and the convergence insufficiency symptom survey (CISS) to discriminate between school-age children with and without CI. The results of that study indicated that the NPC break performed best in identifying children with CI.

However, these results cannot be extended to adults since that study was limited to patients aged 9 to 18 years and because of the limited validity of the Convergence Insufficiency Symptom Survey (CISS) used for children [10]. A previous study used ROC analysis to investigate the diagnostic validity of the clinical signs of CI in participants aged 19 to 35 years with either symptomatic large exophoria and normal heterophoria, or low visual discomfort at near [11]; it reported that NPC and BAF tests showed the best diagnostic accuracy for discriminating between the two groups. In another ROC curve analysis of only BAF and vergence facility (VF) in university students with symptomatic CI and normal participants classified using the CISS [12], BAF showed greater accuracy than VF for diagnosis of CI. All these previous studies have evaluated the validity of diagnostic tests between symptomatic patients with CI and normal participants. However, in addition to cases of independent CI, CI can present with signs associated with other vergence disorders or accommodative disorders in clinical diagnoses of non-strabismic binocular vision anomalies [13]. The symptoms of CI are common in adults, but they often do not appear [1, 5]. Thus, a careful differential diagnosis is required to identify this condition.

The first consideration for screening CI in the presence of non-strabismic binocular vision anomalies is to distinguish it from accommodative dysfunction through analysis of the values or signs measured by each test for accommodative and vergence functions used in the evaluation of binocular vision. The second approach involves identification of CI-related signs. The final approach requires identification of a close correlation between symptoms and clinical signs. In the final approach, however, the symptoms may not be related to clinical signs, especially in adults. Many symptomatic CI disorders are related to the presence of defects in two or more areas of binocular vision function [13, 14].

Our study was limited to young adults and an approach focused on signs and modified signs such as Sheard's and Percival criterion, in addition to fusional vergence related to ability to maintain single binocular vision. The purpose of this study was to evaluate the accommodative and vergence ability for university students who visited for primary eye care due to ocular discomfort and to analyze the diagnostic ability of each test for screening CI by performing ROC curve analysis, including assessments of sensitivity, specificity, cut-off values, and likelihood ratio.

Materials and methods

Subjects

The participants were 184 university students (age = 18-28 years; mean age = 22.23 ± 2.26 years) who underwent an eye examination due to ocular discomfort. Participating students voluntarily visited a university eye clinic center for primary eye care due to blurred vision, eyestrain, and visual discomfort. This study was approved by the Kangwon National University Institutional Review Board (KWNUIRB-2019-02-001-002), with waiver of the informed consent for the retrospective collection of clinical data, and adhered to the tenets of the Declaration of Helsinki. Participants had not previously undergone any vison therapy or eye exercise treatment. We excluded patients with ocular diseases such as glaucoma, cataract, and retinal disease, and those with a history of prior surgery, which was determined by history-taking [1]. The criteria for inclusion into study also were the absence of amblyopia and strabismus.

General procedure

Before refraction assessments, all participants underwent a case evaluation to obtain

information about the ocular discomfort, followed by ocular motility testing and a cover-uncover test to rule out strabismus. Refraction and binocular vision tests were performed using a phoropter (VT-SE; Topcon Corporation, Tokyo, Japan) and visual charts (ACP-8; Topcon Corporation, Tokyo, Japan) at distance (6 m) and near (40 cm). All measurements were taken in a general clinical room by the same examiner, who performed all tests within approximately 30 minutes, using the same methodology.

Assessment of binocular vison function

Following pre-refraction and the best-corrected refraction of ≥20/20 visual acuity, the NPC was measured in free space by using an isolated 20/50 target on a Gulden fixation stick to evaluate the subjects' ability to converge the eyes while retaining binocular single vision [15]. The measured value was determined by the distance (cm) from the subject's eye or spectacle plane at which the subject reported double vision when the target was moved at a rate of approximately 5 cm/s toward the subject between both eyes and at eye level. The normative value for the break point of NPC is 5 ± 2.5 cm.

Phoria was measured by using the von Graefe technique [16] to assess the direction and amplitude of eye alignment, which indicated latent misalignment of the eyes. Horizontal phoria was measured with a measuring prism of 12 Δ (prism) base in (BI) and a dissociating prism of 6 Δ base up (BU) before one eye and the fellow eye, respectively. The measured value was the amount and direction of the prism when the measuring prism of 12 Δ BI was reduced at a rate of approximately 2 Δ/s until the subject reported vertical alignment of the lower and upper 20/30 letter target separated by the two prisms. After measurement of phoria at distance, near phoria was

measured. Negative values represent exophoria, whereas positive values represent esophoria. The normative values for distance phoria and near phoria are 1exo ± 1 Δ and 3exo ± 3 Δ, respectively.

Binocular AA is the maximum amount of accommodation under binocular conditions, which represents the visual function required for maintaining a clear image [17]. AA was measured by the push-up method by using an accommodative convergence rule (GR50, Bernell, USA) and a near target (near visual acuity, 20/30). The near point of accommodation was determined by assessing the distance from the subject's eye or spectacle plane to the target at which the subject reported when the target first became blurred while moving at a rate of approximately 2 cm/s towards the subject's eyes. The amount of AA was expressed in diopter (D) by the inverse of the near point of accommodation (m). The normative value for mean AA is 18.5 − (1/3) age.

Fusional vergences were measured by using the rotary prism on the phoropter and a 20/30 letter target to evaluate the subject's ability to use horizontal vergence to maintain binocular vision. First, negative fusional vergences (NFVs) at distance were measured by using the BI prism [17]. The prism power was increased in front of both eyes at the rate of 1–2 Δ/sec until subjects reported a blurred image (blur point) and then a double image (break point), and then decreased to a find a recovery point until the subject reported a single image. PFVs at distance were measured by base out (BO) prism with the same method as NFVs. Fusional vergences at near were measured after fusional vergence tests at distance [17]. If no blur value in fusional vergence was reported, the break value was used in the analysis. The normative values are 9 ± 2 Δ for bur, 19 ± 4 Δ for break, and 10 ± 2 Δ for recovery of PFV at distance; 7 ± 2 Δ for break, and 4 ± 1 Δ for recovery of NFV at distance; 17 ± 3 Δ for bur, 21 ± 3 Δ for

break, and 11 ± 4 Δ for recovery of PFV at near; 13 ± 2 Δ for bur, 21 ± 2 Δ for break, and 13 ± 3 Δ for recovery of NFV at near.

Gradient AC/A ratio (AC/A) was determined as the difference in phoria between before and after the addition of + 1.00 D at near divided by 1.00 D. Calculated AC/A was determined as the sum of inter-pupillary distance (cm), measured by PD meter, and the difference in phoria between near and distance divided by 2.50 D [17, 18]. The normative value for AC/A is 4 ± 1 Δ.

Relative accommodations were measured to examine the subject's ability to increase and decrease accommodation under binocular vision when the convergence demand was constant [19]. Negative relative accommodation (NRA) was measured first, adding plus power over the refraction at the rate of 0.25 D/2 s. The measured values were the amount of plus power added until the subject reported the first maintained blur. Positive relative accommodation (PRA) was measured as the minus power added over the refraction until the sustained blur. The normative values for NRA and PRA are + 2.00 ± 0.25 D and −2.37 ± 0.62 D, respectively.

BAF was measured at 40 cm by using a ± 2.00 D binocular flipper lens to evaluate the ability of the accommodative response at near [20]. If the subject reported clear vision for a 20/30 letter target when +2.00 D was placed in both eyes, the lens was flipped to place -2.00 D in both eyes. When the subject reported clear vision again, this indicated one cycle. Measured values were in terms of the number of cycles per minute (cpm). The normative value for BAF is > 13 cpm.

Vergence facility (VF) was measured by using a prism flipper (3 Δ BI + 12 Δ BO) to evaluate the ability of the fusional vergence at near (40 cm) [21]. If the subject reported single vision with a 20/30 letter target when 12 Δ BO was placed in both eyes, the prism was flipped to place the 3 Δ BI in both eyes. When the subject

reported clear vision again, this indicated one cycle. Measured values were in terms of the number of cycles per minute (cpm). The normative value for VF is > 12 cpm.

The relation of fusional vergence to phoria was analyzed by using Sheard's [22] and Percival's criteria [23]. Sheard's criterion aimed to determine whether the blur point of fusional vergence is at least twice the phoria. The values for Sheard's criterion were calculated as 2/3 the phoria minus 1/3 the fusional vergence. Vergence anomalies were considered to exist when the subjects failed to meet this criterion (value of Sheard's criterion > 0). Percival's criterion was that the orthophoria point of subject should be operating in the middle-third of the binocular vergence range. The values for Percival's criterion were determined by calculating the value 1/3 greater of two fusional vergences minus 2/3 lesser of the two fusional vergences. If the obtained value was positive, vergence anomalies were considered to be present.

All data were compared with Morgan's expected findings [17, 24], and the main characteristic signs were identified to diagnose accommodative and vergence anomalies. The diagnosis of accommodative and vergence anomalies was classified by signs based on Table 1, which referred to Scheiman and Wick's study [17], and compared with the expected criteria for each test [24]. Subjects groups were classified into three groups according to Table 1: All CI including CI with two signs and CI with an accommodative dysfunction, other binocular vision anomaly (BVA) except CI, and normal binocular vision (NBV). All CI were also classified into CI with three signs (CI3), CI with two signs and an accommodative dysfunction (CI3AD), and CI with two signs (CI2).

Table 1. Diagnostic criteria for non-strabismic binocular vision anomalies.

**Convergence insufficiency (CI)**	**Basic esophoria (BE)**
Presence of sign 1 and sign 2 or 3	Presence of sign 1 and sign 2 or 3
1. Exophoria at near ≥ 4 Δ than at distance	1. Esophoria < 4 Δ difference between distance and near
2. PFV at near < 14 Δ for blur or <18 Δ for break	2. NFV at distance < 5 Δ for break & NFV at near
3. NPC ≥ 7.5 cm	

Divergence insufficiency (DI)
Presence of sign 1 and sign 2 or 3
1. Esophoria at distance ≥ 4 Δ than at near
2. NFV at distance < 5 Δ for break
3. Gradient or calculated AC/A ratio < 3/1

Convergence excess (CE)
Presence of sign 1 and sign 2 or 3
1. Esophoria at near ≥ 4 Δ than at distance
2. NFV at near < 11 Δ for blur or <19 Δ for break
3. Gradient or calculated AC/A ratio > 5/1

Divergence excess (DE)
Presence of sign 1 and sign 2 or 3
1. Exophoria at distance ≥ 4 Δ than at near
2. PFV at distance < 7 Δ for blur or <15 Δ for break
3. Gradient or calculated AC/A ratio > 5/1

Basic exophoria (BX)
Presence of sign 1 and sign 2 or 3
1. Exophoria < 4 Δ difference between distance and near
2. PFV at distance < 7 Δ for blur or <15 Δ for break, and PFV at near < 14 Δ for blur or <18 Δ for break
3. Gradient or calculated AC/A ratio 3–5/1

< 11 Δ for blur or <19 Δ for break
3. Gradient or calculated AC/A ratio 3–5/1

Fusional vergence dysfunctions (FVD)
Presence of sign 1 and sign 2 or 3
1. Normal phoria at distance (1 exo ± 1Δ) and near (3 exo ± 3Δ)
2. Reduced (low) PFV and NFV at distance and near
3. Gradient or calculated AC/A ratio 3–5/1

Accommodative insufficiency (AI)
Presence of sign 1 and sign 2 or 3
1. Reduced binocular AA for minimum amplitude = 15.0 - 0.25 × age
2. Low PRA < 1.75 D
3. BAF < 13 cpm

Accommodative excess (AE)
Presence of signs 1 and 2
1. Low NRA < 1.75 D
2. BAF < 13 cpm

Accommodative infacility
Presence of signs 1 and 2
1. Low NRA & PRA < 1.75 D
2. BAF < 13 cpm

D: diopter, Δ: prism diopter, cpm: cycles per minute, AA: accommodative amplitude, NPC: near point of convergence, AC/A: accommodative convergence/accommodation, NFV: negative fusional vergence, PFV: positive fusional vergence, NRA: negative relative accommodation, PRA: positive relative accommodation, BAF: binocular accommodative facility.

Data analysis

Data were collected and analyzed using IBM SPSS Statistics version 19 (IBM Corp., USA). One-way ANOVA with a Bonferroni's *post-hoc* test comparing the three groups (all CI, BVA, and NBV) was performed using mean and standard deviation values. A p-value of < 0.05 was considered significant. ROC curve analysis was performed by plotting sensitivity on the y axis as a function of 1 – specificity on the x axis to analyze the diagnostic ability of each test (AA, NPC, AC/A, NFV, PFV, NRA, PRA, BAF, VF, Sheard's and Percival's criterion) in screening CI. Sensitivity refers to the probability that a test will indicate CI when CI is present, and specificity refers to the probability that a test will indicate the absence of CI when CI is not present.

The area under the curve (AUC) in the ROC curve analysis indicates the discriminative ability to distinguish between subjects with and without CI. The cut-off value for each test was defined as the coordinate that had the maximized sum of sensitivity and specificity. The cut-off was also identified for each test with the largest AUC which was significantly greater than 0.50. The likelihood ratio indicates the degree of increase or decrease in the probability of the CI if the test yields positive or negative findings.

Results

In an assessment of the refractive errors in the participants, of the 368 eyes, 258 (70.1%) showed myopia (spherical equivalent [SE] = -3.31 ± 2.16 D), 19 (5.2%) showed hyperopia (SE = +0.80 ± 0.51 D), and 91 (24.7%) showed emmetropia. Myopia was present in 125 participants (67.9%), hyperopia in six participants (3.3%), emmetropia in 42 participants (22.8%), mixed anisometropia in four participants (2.2%), simple myopic anisometropia in four participants (2.2%), and simple hyperopic anisometropia in three participants (1.6%).

The means and standard deviations for each test of binocular vision function after wearing refractive correction are shown in Table 2. Subjects were divided into three groups: all CI (n = 39); binocular vision anomalies (BVAs; n = 49) except CI; and normal binocular vision (NBV; n = 96). The one-way ANOVA with Bonferroni's *post-hoc* test showed significant intergroup differences in NPC, near phoria, and calculated AC/A; significant differences between all CI and other BVA, and other BVA and NBV in distance phoria and distance NFV; and significant differences between all CI and NBV in gradient AC/A, PFV blur and recovery at distance, near PFV, NRA, PRA, BAF, and VF.

Table 2. Comparison of mean and standard deviation values for the measures of binocular function.

Test	All subjects (n = 184)	Subject groups			
		a. All CI (n = 39)	b. Other BVA (n = 49)	c. NBV (n = 96)	p-value (*post-hoc*)
AA (D)	11.46 ± 2.48	10.90 ± 1.75	11.24 ± 2.83	11.79 ± 2.51	0.130
NPC (cm)	7.29 ± 1.93	9.21 ±2.07	7.24 ± 1.85	6.53 ± 1.27	< 0.001* (a>b>c)
Phoria at distance (Δ)†	-2.28 ± 2.91	-3.04 ± 2.10	-0.88 ± 4.04	-2.69 ± 2.20	< 0.001* (a, c>b)
Phoria at near (Δ)†	-6.34 ± 5.63	-10.37 ± 2.84	-1.71 ± 5.93	-7.10 ± 4.63	< 0.001* (a>c>b)
Calculated AC/A (Δ/D)	4.82 ± 1.82	3.55 ± 1.33	6.16 ± 2.06	4.66 ± 1.38	< 0.001* (b>c>a)
Gradient AC/A (Δ/D)	3.14 ± 1.62	2.40 ± 1.42	3.54 ± 1.73	3.23 ± 1.55	< 0.003* (b, c>a)
NFV break at distance (Δ)	9.11 ± 3.99	9.72 ± 3.80	7.78 ± 3.64	9.54 ± 4.12	0.023* (a, c>b)
NFV recovery at distance (Δ)	5.28 ± 2.91	6.15 ± 3.10	4.25 ± 2.45	5.46 ± 2.92	0.006* (a, c>b)
PFV blur at distance (Δ)	10.08 ± 4.51	8.69 ± 3.18	9.02 ± 3.95	11.19 ± 4.97	0.002* (c>a, b)
PFV break at distance (Δ)	16.16 ± 7.84	14.62 ± 5.95	14.04 ± 7.40	17.87 ± 8.39	0.008* (c>b)
PFV recovery at distance (Δ)	9.76 ± 6.57	8.03 ± 4.68	8.55 ± 5.87	11.08 ± 7.30	0.015* (c>a)
NFV blur at near (Δ)	13.47 ± 5.19	14.10 ± 5.00	12.10 ± 5.72	13.91 ± 4.91	0.097
NFV break at near (Δ)	19.92 ± 6.55	21.00 ± 5.67	18.25 ± 7.84	20.33 ± 6.05	0.097
NFV recovery at near (Δ)	13.51 ± 5.85	14.03 ± 4.99	12.47 ± 6.80	13.82 ± 5.65	0.348
PFV blur at near (Δ)	15.52 ± 6.23	13.41 ± 6.41	13.41 ± 6.36	17.45 ± 5.47	< 0.001* (c>a, b)
PFV break at near (Δ)	21.15 ± 8.48	17.92 ± 8.10	19.08 ± 8.63	23.51 ± 7.90	< 0.001* (c>a, b)
PFV recovery at near (Δ)	13.39 ± 8.70	11.10 ± 8.71	11.43 ± 7.61	15.32 ± 8.85	0.006* (c>a, b)
NRA (D)	2.15 ± 0.63	1.87 ± 0.65	1.92 ± 0.65	2.39 ± 0.50	< 0.001* (c>a, b)
PRA (D)	-2.77 ± 1.15	-2.76 ± 1.26	-2.40 ± 1.11	-2.98 ± 1.10	0.017* (c>b)
BAF (cpm)	12.47 ± 5.64	10.74 ± 5.61	12.12 ± 6.63	13.35 ± 4.95	0.044* (c>a)
VF (cpm)	13.27 ± 3.93	10.59 ± 4.09	13.61 ± 4.04	14.19 ± 3.31	< 0.001* (c>a)
Sheard's criterion at distance	-1.22 ± 2.00	-0.87 ± 1.80	-0.30 ± 2.15	-1.83 ± 1.80	< 0.001* (a, b>c)
Sheard's criterion at near	-0.15 ± 3.25	2.44 ± 2.75	-0.69 ± 2.98	-0.92 ± 3.05	< 0.001* (a>b, c)
Percival's criterion at distance	-1.11 ± 1.69	-1.27 ± 1.39	-0.54 ± 1.60	-1.32 ± 1.80	0.023 (b>c)
Percival's criterion at near	-1.77 ± 2.34	-1.11 ± 2.32	-1.23 ± 2.34	-2.32 ± 2.23	0.004 (a, b>c)

CI: convergence insufficiency, BVA: binocular vision anomaly, NBV: normal binocular vision, D: diopter, Δ: prism diopter, cpm: cycles per minute, AA: accommodative amplitude, NPC: near point of convergence, AC/A: accommodative convergence/accommodation, NFV: negative fusional vergence, PFV: positive fusional vergence, NRA: negative relative accommodation, PRA: positive relative accommodation, BAF: binocular accommodative facility, VF: vergence facility. $^{*}p < 0.05$ indicates statistically significant differences among groups in one-way ANOVA followed by Bonferroni's *post-hoc* test. †Minus and plus signs in phoria indicate exophoria and esophoria, respectively.

Table 3 shows the prevalence of binocular vision anomalies diagnosed according to Table 1 based on data for each test prior to the ROC curve analysis. It also shows the characteristics of the subjects enrolled in CI screening assessments. Of these 184 young adults, 96 (52.2%) were classified as NBV and 34 (18.5%) were identified as

having CI2. CI with accommodative disorders (CI + AI and CI + AE) was observed in five (2.7%) participants, while the prevalence of CI varied from 10.3% (19 participants with CI3) to 21.2% (39 participants with CI2AD) depending on the number of signs and the presence of associated accommodative disorders. When participants with BVAs other than CI were referred to as non-CI, the distribution between CI and non-CI was different according to the classification criteria.

Table 3. Prevalence of binocular vision anomalies.

Dysfunction	n	%	CI subjects associated with signs and AD
CI	34	18.5	
DI	11	6.0	
CE	4	2.2	
DE	2	1.1	Classified CI vs. non-CI group†
BX	9	4.9	
BE	4	2.2	CI + 3 signs (CI3): 19 (10.3%) vs. 165 (89.7%)
AI	7	3.8	
AE	2	1.1	CI + 3 signs + AD (CI3AD): 23 (12.5%) vs. 161 (87.5%)
CI + AI	4	2.2	
CI + AE	1	0.5	CI + 2 signs (CI2): 34 (18.5%) vs. 150 (81.5%)
CE + AE	3	1.6	
BX + AI	1	0.5	CI + 2 signs + AD (CI2AD): 39 (21.2%) vs. 145 (78.8%)
BE + AI	1	0.5	
Indefinite	5	2.7	
NBV	96	52.2	
Total	184	100	

Abbreviations are the same as those in the notes for Table 1. Signs are sign 1, 2, 3 for CI in Table 1. AD: accommodative dysfunction, CI3: CI with 3 signs, CI3AD: CI with 3 signs + CI with an accommodative dysfunction, CI2: CI with 2 signs, CI2AD: CI with 2 signs + CI with an accommodative dysfunction. †Non-CI group is a group excluding CI group.

ROC curve analysis was performed for the tests shown in Table 2. The results of the AUC for NPC, Sheard's and Percival's criterion with $p < 0.05$ and AUC > 0.5 in 95% confidence interval ranges, and PFV, NFV, and AC/A including the diagnostic criteria in Table 1 are shown in Table 4. Fig 1 shows ROC curves for NPC, Sheard's and Percival's criterion with statistically significant differences when comparing the AUC to the value 0.5. Among the AUCs for all diagnostic tests included in Table 1, the values were significantly greater than 0.5 for only the NPC and NFV at distance,

and the AUCs for diagnostic tests excluded in Table 1 were also significantly greater than 0.5 for Sheard's and Percival's criteria. AUCs were greater for NBV than for non-CI, greater for excluding than for including CI associated with AD, and greater for CI with three signs than for CI with two signs.

Table 4. Results for the area under the curve (AUC) in ROC curve analysis among CI, non-CI, and NBV groups.

Ability to discriminate CI	NPC	Sheard[†]	Percival[†]	NFV	PFV[ns]	AC/A[ns]
From CI3 + NBV	0.920	0.912	0.717	(0.627) rec	< 0.2	< 0.3
From CI3 + non-CI	0.842	0.905	0.672	(0.654) rec	< 0.2	< 0.3
From CI3AD + NBV	0.920	0.905	0.702	0.577[ns] brk	< 0.2	< 0.3
From CI3AD + non-CI	0.853	0.900	0.657	0.601[ns] brk	< 0.2	< 0.3
From CI2 + NBV	0.914	0.793	0.652	(0.614) rec	< 0.4	< 0.3
From CI2 + non-CI	0.866	0.773	0.608	(0.656) rec	< 0.5	< 0.3
From CI2AD + NBV	0.913	0.804	0.642	(0.566)[ns] rec	< 0.4	< 0.4
From CI2AD + non-CI	0.876	0.795	0.598	(0.603) rec	< 0.4	< 0.4

Abbreviations are the same as those in the notes for Tables 1, 2, and 3. [†]Criterion for near, brk: break point, rec: recovery point, [ns]: not significant, ( ): value for distance.

Fig 1. ROC curves for NPC and Sheard's and Percival's criterion according to the combined CI groups.

Blue solid line: NPC, red dotted line: Sheard's criterion for near, dashed line: Percival's criterion for near, diagonal line: no discrimination (AUC = 0.5).

Table 5 shows the sensitivity, specificity, positive likelihood ratio (LR+), and negative likelihood ratio (LR-) for each test by using cut-offs obtained with ROC curves. The NPC cut-offs were >7.2 cm for all classified CIs combined with the NBV and non-CI groups. The Sheard's criterion cut-off of >1.00 for CI with three signs (CI3) was higher than the cut-off of >-0.23 for CI with two signs (CI2), and showed higher sensitivity and specificity than CI2. The Percival criterion cut-off of >-4.00 for

CI2 was lower than the cut-off of >-2.33 for CI3. Sensitivity, specificity, and LR+ were higher in the order of NPC, Sheard's, and Percival's criterion, but LR- showed the opposite trend.

Table 5. Diagnostic validity of NPC and Sheard's and Percival's criterion by using cut-offs derived from ROC curve analysis

Screening for combined CI groups			Cut-off	Sensitivity	Specificity	LR+	LR-
CI3	+ NBV	NPC	>7.20	1.00	0.84	6.40	0.00
		Sheard	>0.33	1.00	0.74	3.84	0.00
		Percival	>-2.33	0.84	0.56	1.92	0.28
	+ non-CI	NPC	>7.20	1.00	0.68	3.11	0.00
		Sheard	>0.33	1.00	0.67	3.06	0.00
		Percival	>-2.33	0.84	0.47	1.60	0.33
CI3AD	+ NBV	NPC	>7.20	1.00	0.84	6.40	0.00
		Sheard	>0.33	1.00	0.74	3.84	0.00
		Percival	>-2.33	0.83	0.56	1.89	0.31
	+ non-CI	NPC	>7.20	1.00	0.70	3.29	0.00
		Sheard	>1.00	0.96	0.74	3.67	0.06
		Percival	>-2.33	0.83	0.48	1.58	0.36
CI2	+ NBV	NPC	>7.20	1.00	0.84	6.40	0.00
		Sheard	>-0.23	0.82	0.69	2.64	0.26
		Percival	>-2.33	0.71	0.56	1.61	0.52
	+ non-CI	NPC	>7.20	1.00	0.75	3.95	0.00
		Sheard	>-0.23	0.82	0.63	2.25	0.28
		Percival	>-4.00	0.97	0.24	1.28	0.12
CI2AD	+ NBV	NPC	>7.20	1.00	0.84	6.40	0.00
		Sheard	>-0.23	0.85	0.69	2.71	0.22
		Percival	>-4.00	0.97	0.28	1.36	0.09
	+ non-CI	NPC	>7.20	1.00	0.77	4.39	0.00
		Sheard	>-0.23	0.85	0.66	2.45	0.23
		Percival	>-4.00	0.97	0.25	1.30	0.10

Abbreviations are the same as those in the notes for Tables 1, 2, and 3. LR+: positive likelihood ratio, LR-: negative likelihood ratio.

Discussion

In this study, with ROC curve analysis of signs, the tests that showed a significant discriminative ability between CI and non-CI or CI and NBV among young adult university students were those based on the Sheard's and Percival's criteria for near vision, while NPC assessment was the best diagnostic test for identifying CI. For CI

screening between CI3 (CI with three signs) and non-CI, Sheard's criterion was a better diagnostic parameter than NPC. The distribution of CI, non-CI, and NBV according to diagnostic criteria such as population and the signs of CI influenced the validity of each test in the ROC curve analysis.

The prevalence of CI in this study ranged between 10.3% and 21.2%, higher than rates of 1.5% to 10.8% reported in previous studies of the general population–university students [8, 25, 26]. These variations in the prevalence of CI can be attributed to differences in methodological aspects, including instrumentation and techniques, classification criteria, and the number of diagnostic signs; the types of populations studied (clinical/non-clinical); data analysis methods; and participant factors including age as well as refractive errors [27]. Our study was performed in university students who underwent primary eye care due to ocular discomfort, and CI was classified based on signs. The CI screening ability of each test was also evaluated in NBV and non-CI conditions. The variation in CI prevalence in this study appears to be associated with differences in signs, classification criteria, and population. In our study, the prevalence of myopia was high (70.1%). CI has been reported to show a significant association with myopia [28], whereas CI and refractive errors were not significantly associated [29]. Although two previous studies showed different results, the high prevalence of CI in this study may be partially explained by the association with myopia. CI may present as types associated with accommodative dysfunction such as CI plus AI [30] or CI plus accommodative excess (AE) [19]. CI combined with accommodative functions can also exist because the vergence and accommodative systems are linked [31]. CI should also be distinguished from conditions such as normal vision, diverse vergence and accommodative disorders, and other binocular anomalies. Our study factored these conditions to investigate the

discriminative ability of each test for CI screening by performing ROC curve analysis under these conditions.

In comparisons of the means and standard deviations for each test, assessments based on NPC, AC/A, PFV blur/break/recovery at near, NRA, BAF, and VF showed significant differences between all-CI and NBV groups, and those based on NPC and AC/A showed significant differences between all-CI and other BVA groups. Tests with a significant difference were noted less frequently in comparisons with other BVA than in comparisons with NBV, as shown in Table 2. In Cacho-Martínez et al.'s [11] study comparing large phoria (>6 Δ) and normal phoria, significant differences were found on 5 tests such as NPC, PFV blur at near, NRA, BAF, and VF. However, in our study comparing all-CI and other BVA groups, significant differences were found on 2 tests of NPC and AC/A. This finding indicates that it is difficult to distinguish CI from other BVA. Since patients with CI must be distinguished from subjects with various other clinical conditions, the efficiency of CI screening depends on the performance of the diagnostic test in discriminating CI from abnormal groups combined with other binocular anomalies rather than normal groups. Tests for the diagnosis of CI were based on the ratio of positive fusional vergence to phoria [9] and Sheard's criterion in other studies [22, 32], and these tests was limited to school-age children. However, Percival's criterion as well as Sheard's criterion was applied to adults in this study. There are significant differences between the all-CI and NBV groups for Sheard's criterion at distance/near and Percival's criterion at near and between the all-CI and other-BVA groups for Sheard's criterion at near. These results indicate that Sheard's criterion could be used in tests to distinguish CI from other BVA and NBV, and Percival's criterion could be used to distinguish CI from NBV. However, Sheard's criterion is a useful tool for screening CI with exophoria

associated with near tasks because the signs of CI include exophoria more than 6 Δ at near and normal phoria of 0–6 Δ exophoria at distance [17], and previous studies [33, 34] have suggested that Sheard's and Percival's criteria are the most effective with exophoria and esophoria, respectively.

The main finding in this study was that NPC can distinguish individuals with CI signs or CI signs associated with AD, namely, CI2AD and CI3AD from the NBV and non-CI groups. In ROC curve analysis, the AUC of 0.842–0.920 obtained using the NPC test represents an excellent discriminative ability for CI screening. Although the test parameters such as subjects and diagnostic and classification criteria were not consistent with other studies [25, 26], our result is consistent with outcomes that suggest that NPC is the best in identifying children with CI [9] and subjects with large near exophoria and moderate to severe symptoms [11]. The discriminative ability in CI groups (CI3, CI3AD, CI2, CI2AD) combined with NBV is higher than that in CI groups combined with non-CI. The NPC test showed a cut-off value of >7.2 cm in comparison with previously reported values of 7 cm [11] or 7.5 cm [35, 36] and sensitivity of 1.00 in all groups, and specificity and positive likelihood ratio (LR+) were higher in the combined group of CI and NBV than in the combined group of CI and non-CI. A high LR+ indicates a high ratio of the probability of the true presence of CI to the probability of false presence of CI in the NPC test. A low LR- indicates a low ratio of the probability of false absence of CI to the probability of true absence of CI in the NPC test. A negative likelihood ratio (LR-) of zero in all classified CIs indicates a decreased probability that the NPC test is negative.

The ROC curve analysis in this study showed that Sheard's and Percival's criteria have potential for use as tools for CI screening. Sheard's criterion is particularly useful for CI screening from non-CI than from NBV. In addition, Sheard's criterion

can be a better tool than NPC in cases of CI with three signs (CI3, CI3AD). The previous studies [9, 11] did not provide cut-off values, sensitivity, specificity, AUC, and other data from ROC curve analysis for Sheard's and Percival's criterion to screen CI. The AUC of 0.773– 0.912 obtained using Sheard's criterion in our study represents an acceptable discriminative ability for CI screening, and the AUC reduced with decreasing signs. Sheard's criterion in this study has positive cut-offs values (failed to normal binocular vision or needed prism) in the CI3 and cut-offs of > -0.23 (approximately cut-offs > zero) in the CI2. Sheard's criterion could diagnose all-CI. The sensitivity of the combined CI3 is higher than that of the combined CI2, and the specificity is lower than sensitivity. Although the LR+ of 2.25–3.84 was lower than the corresponding value for the NPC assessment as a positive test result indicating the presence of CI, and the LR- of 0.22–0.28 was higher than the corresponding value for the NPC assessment as a negative result test indicating the absence of CI, from another perspective, this criterion is a valid tool for discriminating CI with three signs (CI3, CI3AD) from non-CI because the AUC of Sheard's criterion was greater than that in NPC and the LR- of zero in CI3 and CI3AD was equal to that for NPC.

Percival's criterion showed cut-off values of <0 (negative value; meet criterion or not needed prism) in all groups. Although Percival's criterion showed cut-off values for screening CI, the ROC curve analysis values indicate that it is a valid tool for CI screening when considering AUC ≥ 0.598, sensitivity ≥ 0.71, specificity of 0.24 – 0.56, LR+ ≥ 1.28, and LR- ≤ 0.36. Values of for CI screening showed a mismatch between Percival's criterion (the amount of prism required or a positive cut-off value) and the results of ROC curve analysis (a negative cut-off value) but an approximate match between Sheard's criterion (the amount of prism required or a positive value) and the results of ROC curve analysis (cut-off value of -0.23 close to a positive).

These differences could have occurred because Sheard's criterion works best for exophoric conditions such as CI and Percival's criterion tends to work best for near esophoric conditions such as convergence excess [23, 37]. However, Percival's criterion showed a lower discriminative ability than Sheard's criterion for CI screening. In ROC curve analysis, Percival's criterion showed lower AUC and LR+ than Sheard's criterion. Percival's criterion also showed lower sensitivity than Sheard's criterion, except for CI2 combined with non-CI (CI2 + non-CI) and CI2 combined with AD (CI2AD + NBV, CI2AD + non-CI). The LR- of Percival's criterion was higher than that of Sheard's criterion except for CI2 combined with non-CI and combined CI2AD. Although Percival's criterion could be a useful tool for CI screening, this criterion needs to be modified to show better diagnostic accuracy for binocular vision disorders. The ROC curve analysis for each test except the above showed no statistically significant differences for CI screening, but significant differences were noted for BAF in other studies [9, 12]. On the other hand, a high PRA was shown to be the most sensitive sign for CI combined with accommodative excess [19], and Gall at al. [21] found that VF can differentiate symptomatic from asymptomatic patients (not assessed in this study). However, BAF, PRA, and the VF test were not shown to be useful in discriminating CI from the normal or non-CI groups in the results of our study. Thus, factors related to the classification criteria for binocular vision disorders and subject characteristics such as age and population might lead to a different result.

Although this study was conducted with participants reporting ocular discomfort, one limitation of this study is the lack of evaluation about subjective symptoms since the analysis was based on objective clinical signs. In the previous studies, some adults with CI signs were asymptomatic [38] and there was no further association between

the severity of the clinical signs and symptoms in children aged 9 to 17 years [4]. On the other hand, other studies have shown an association between signs and symptoms [9, 39]. These results cannot extend to young adults. Therefore, the relationship between signs and symptoms in university students needs further study.

In summary, this study shows that Sheard's and Percival's criteria are useful tools to discriminate CI in young adults although the NPC test has diagnostic validity for screening subjects with CI signs from not only NBV but also non-CI with AD and other binocular vision disorders. On the other hand, with respect to the AUCs of the ROC curve analysis for CI screening in cases of CI with three signs, Sheard's criteria were significantly greater than NPC and Percival's criteria. In addition, this study suggests that Percival's criterion such as "orthophoria point should be in the middle third of the vergence range" needs to be revised due to showing acceptable sensitivity and specificity for CI screening at a negative cut-off value within the range to meet this criterion.

Supporting Information

S1 File. All relevant raw data.

(XLSX)

Acknowledgements

Not applicable.

References

1. Cooper JS, Burns CR, Cotter SA, Daum KM, Griffin JR, Scheiman MM. Optometric clinical practice guidelines: care of the patient with accommodative and vergence dysfunctions. St Louis: American Optometric Association; 2010. pp. 4-23. https://www.aoa.org/documents/optometrists/CPG-18.pdf

2. Wick BC. Horizontal deviations. In: Amos JF, ed. Diagnosis and management in vision care. Boston: Butterworth-Heinemann; 1987. pp. 461-510.

3. García-Muñoz Á, Carbonell-Bonete S, Cacho-Martínez P. Symptomatology associated with accommodative and binocular vision anomalies. J Optom. 2014; 7: 178-192. doi: 10.1016/j.optom.2014.06.005

4. Bade A, Boas M, Gallaway M, Mitchell GL, Scheiman M, Kulp MT, et al. Relationship between clinical signs and symptoms of convergence insufficiency. Optom Vis Sci. 2013; 90: 988-995. doi: 10.1097/OPX.0000000000000012

5. Cooper J, Jamal N. Convergence insufficiency-a major review. Optometry. 2012; 83: 137-158. PMID: 23231437

6. Dwyer P. The prevalence of vergence accommodation disorders in a school-age population. Clin Exp Optom. 1992; 75: 10-18. doi: 10.1111/j.1444-0938.1992.tb01010.x

7. Paniccia SM, Ayala AR. Prevalence of accommodative and non-strabismic binocular anomalies in a Puerto Rican pediatric population. Optom Vis Perform. 2015; 3: 158-164. https://www.ovpjournal.org/uploads/2/3/8/9/23898265/ovp3-3_article_paniccia_web.pdf

8. Cacho-Martínez P, García-Muñoz Á, Ruiz-Cantero MT. Do we really know the prevalence of accomodative and nonstrabismic binocular dysfunctions? J Optom. 2010; 3: 185-197. doi: 10.1016/S1888-4296(10)70028-5

9. Menjivar AM, Kulp MT, Mitchell GL, Toole AJ, Reuter K. Screening for convergence insufficiency in school-age children. Clin Exp Optom 2018; 101: 578-584. doi: 10.1111/cxo.12661

10. Rouse M, Borsting E, Mitchell GL, Cotter SA, Kulp M, Scheiman M, et al. Validity of the convergence insufficiency symptom survey: a confirmatory study. Optom Vis Sci. 2009; 86: 357-363. doi: 10.1097/OPX.0b013e3181989252

11. Cacho-Martínez P, García-Muñoz A, Ruiz-Cantero MT. Diagnostic validity of clinical signs associated with a large exophoria at near. J Ophthalmol. 2013; 2013: 549435. doi: 10.1155/2013/549435

12. Kim YH. Binocular accommodative facility and vergence facility in university students with symptomatic convergence insufficiency. J Korean Ophthalmic Opt Soc**.** 2018; 23: 117-123**.** doi: 10.14479/jkoos.2018.23.2.117

13. Darko-Takyi C, Khan NE, Nirghin U. A review of the classification of nonstrabismic binocular vision anomalies. Optometry Reports. 2016; 5: 5626. doi: 10.4081/optometry.2016.5626

14. Cacho Martínez P, García Muñoz A, Ruiz-Cantero MT. Treatment of accommodative and nonstrabismic binocular dysfunctions: a systematic review. Optometry. 2009; 80: 702-716. doi: 10.1016/j.optm.2009.06.011

15. Scheiman M, Gallaway M, Frantz KA, Peters RJ, Hatch S, Cuff M, et al. Nearpoint of convergence: test procedure, target selection, and normative data. Optom Vis Sci. 2003; 80: 214-225. doi: 10.1097/00006324-200303000-00011

16. Maples WC, Savoy RS, Harville BJ, Golden LR, Hoenes R. Comparison of distance and near heterophoria by two clinical methods. Optom Vis Dev. 2009; 40: 100-106. https://cdn.ymaws.com/www.covd.org/resource/resmgr/ovd40-2/article_comparisonofdistance.pdf
17. Scheiman M. Wick B. Clinical management of binocular vision: heterophoric, accommodative, and eye movement disorders, 4th ed. Philadelphia: Lippincott Williams & Wilkins; 2014. pp. 36-76.
18. Tsai LH, Lin DPC, Su JKC, Chang YS, Chen ST. The effects of myopia and AC/A measuring methodology on AC/A ratio outcome. Chung Shan Medical Journal. 2012; 23: 87-94. doi: 10.30096/CSMJ.201212.0003
19. García A, Cacho P, Lara F. Evaluating relative accommodations in general binocular dysfunctions. Optom Vis Sci. 2002; 79: 779-787. doi: 10.1097/00006324-200212000-00010
20. Weissberg EM. Diagnostic techniques. In: Weissberg EW, ed. Essential of clinical binocular vision. St.Louis: Butterworth-Heinemann; 2004. pp. 158-164.
21. Gall R, Wick B, Bedell H. Vergence facility: establishing clinical utility. Optom Vis Sci. 1998; 75: 731-742. doi: 10.1097/00006324-199810000-00018
22. Worrell BE Jr, Hirsch MJ, Morgan MW. An evaluation of prism prescribed by Sheard's criterion. Am J Optom Arch Am Acad Optom. 1971; 48: 373-376. PMID: 4931926
23. Percival A. The prescribing of spectacles, 3rd ed. Bristol: John Wright & Sons; 1928. pp. 102-109. https://archive.org/details/b21287405/page/102
24. Benjamin WJ. Borish’s clinical refraction, 2nd ed. St. Louis: Butterworth-Heinemann; 2006. pp. 968-972.

25. Cooper J, Duckman R. Convergence insufficiency: incidence, diagnosis, and treatment. J Am Optom Assoc. 1978; 49: 673-680. PMID: 355298

26. Porcar E, Martinez-Palomera A. Prevalence of general binocular dysfunctions in a population of university students. Optom Vis Sci. 1997; 74: 111-113. doi: 10.1097/00006324-199702000-00023

27. Wajuihian SO, Hansraj R. A review of non-strabismic accommodative-vergence anomalies in school-age children. Part 1: Vergence anomalies. Afr Vision Eye Health. 2015; 74: 1-10. doi: 10.4102/aveh.v74i1.32

28. Gupta R, Sharma B, Anand R, Bawaria S, Dewada R. Association of asthenopia and convergence insufficiency in children with refractive error - a hospital based study. Int J Med Res Rev. 2013; 1: 222-227. http://medresearch.in/index.php/IJMRR/article/view/51/178

29. Wajuihian SO. Is there an association between convergence insufficiency and refractive errors?. Afr Vision Eye Health. 2017; 76: 1-7. doi: https://doi.org/10.4102/aveh.v76i1.363

30. Davis AL, Harvey EM, Twelker JD, Miller JM, Leonard-Green T, Campus I. Convergence insufficiency, accommodative insufficiency, visual symptoms, and astigmatism in Tohono O'odham students. J Ophthalmol. 2016; 2016: 1-7. doi: 10.1155/2016/6963976

31. Schor CM. A dynamic model of cross-coupling between accommodation and convergence: simulations of step and frequency responses. Optom Vis Sci. 1992; 69: 258-269. doi: 10.1097/00006324-199204000-00002

32. Convergence Insufficiency Treatment Trial Study Group. Randomized clinical trial of treatments for symptomatic convergence insufficiency in children. Arch Ophthalmol. 2008; 126: 1336-1349. doi: 10.1001/archopht.126.10.1336

33. Sheedy JE, Saladin JJ. Association of symptoms with measures of oculomotor deficiencies. Am J Optom Physiol Opt. 1978; 55: 670-676. PMID: 747192

34. O'Leary CI, Evans BJ. Double-masked randomised placebo-controlled trial of the effect of prismatic corrections on rate of reading and the relationship with symptoms. Ophthalmic Physiol Opt. 2006; 26: 555-565. doi: 10.1111/j.1475-1313.2006.00400.x

35. Yu DS, Cho HG, Moon BY. Influence of different diagnostic criteria on frequency of convergence insufficiency. J Korean Ophthalmic Opt Soc. 2016; 21: 235-242. doi: 10.14479/jkoos.2016.21.3.235

36. Gallaway M, Scheiman M, Malhotra K. The effectiveness of pencil pushups treatment for convergence insufficiency: a pilot study. Optom Vis Sci. 2002; 79: 265-267. doi: 10.1097/00006324-200204000-00013

37. Sheedy JE, Saladin JJ. Phoria, vergence, and fixation disparity in oculomotor problems. Am J Optom Physiol Opt. 1977; 54: 474-478. PMID: 931014

38. Horwood AM, Toor S, Riddell PM. Screening for convergence insufficiency using the CISS is not indicated in young adults. Br J Ophthalmol. 2014; 98: 679-683. doi: 10.1136/bjophthalmol-2013-304533

39. Borsting E, Rouse MW, Deland PN, Hovett S, Kimura D, Park M, et al. Association of symptoms and convergence and accommodative insufficiency in school-age children. Optometry. 2003; 74: 25-34. PMID: 12539890

Figure

Click here to access/download;Figure;Fig 1.tif

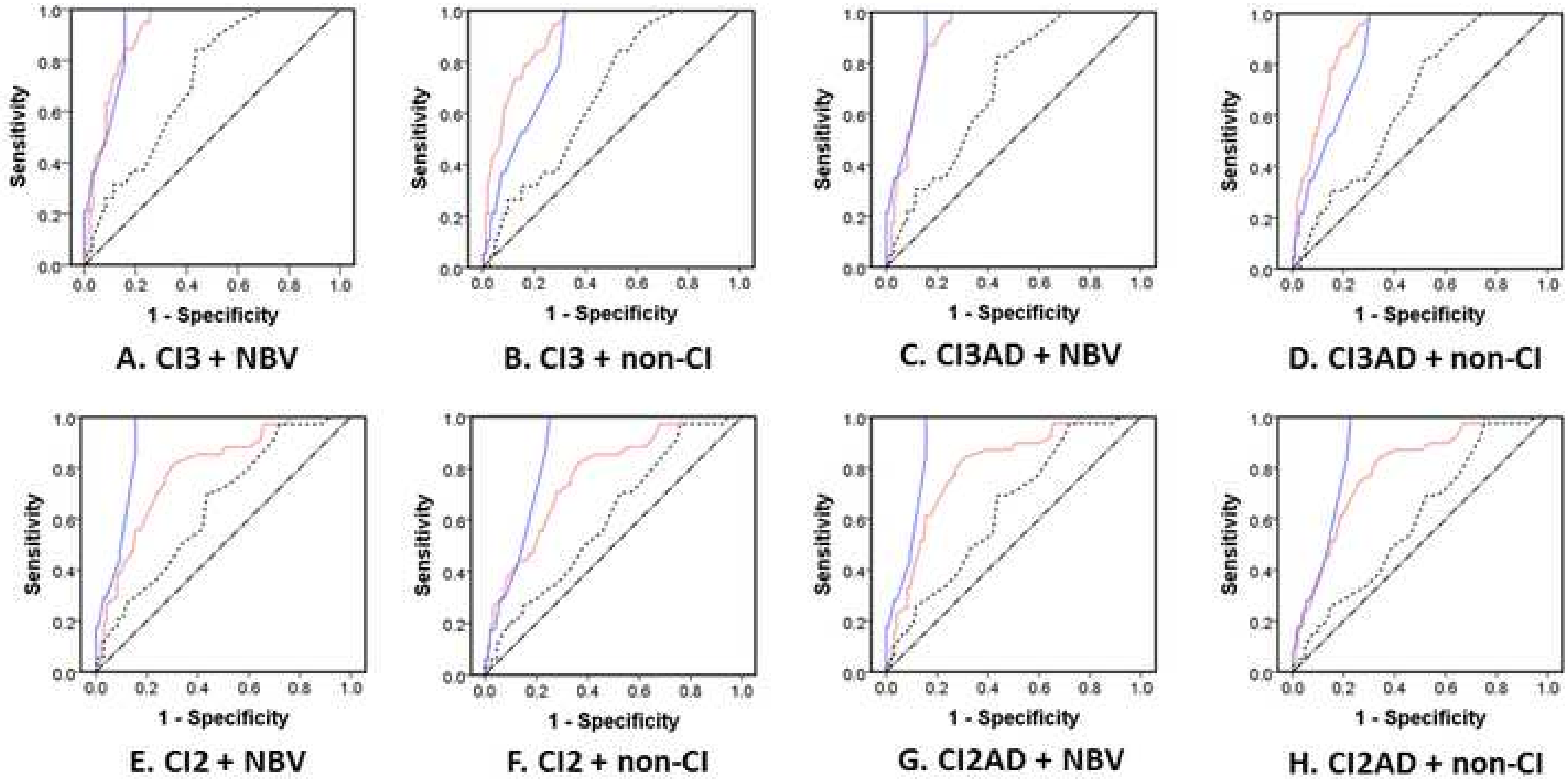

Click here to access/download
Supporting Information
S1 File.xlsx

Receiver operating characteristic curve analysis of clinical signs for screening of convergence insufficiency in young adults

Byeong-Yeon Moon[1], Sang-Yeob Kim[1], Dong-Sik Yu[1]*

Formatted: Superscript

[1]Department of Optometry, Kangwon National University, Samcheok, Korea

* Corresponding author
E-mail: yds@kangwon.ac.kr

Abstract

Convergence insufficiency (CI) is a common dysfunction of binocular vision disorder that is associated with various signs and symptoms in near work. However, CI screening is performed less frequently in adults than in children. We aimed to evaluate the ability of screening tests to discriminate CI from other binocular vision anomalies and normal binocular vision in young adults. One hundred eighty-four university students (age, 18-28 years) who underwent an eye examination due to ocular discomfort were included. Near point of convergence (NPC), phoria, accommodative amplitude, fusional vergence, the ratio of accommodative convergence to accommodation, relative accommodation, binocular accommodative facility, vergence facility, and the values corresponding to Sheard's and Percival's criteria were evaluated. Receiver operating characteristic (ROC) curve analysis for each test was also performed. The prevalence of CI ranged from 10.3% to 21.2%, depending on the signs and the presence of CI associated with accommodative disorders. Assessments based on NPC, Sheard's criterion, and Percival's criterion showed high discriminative ability, with the ability being higher between the CI and normal binocular vision groups than between the CI and non-CI groups. Sheard's criterion showed the highest diagnostic performance in discriminating CI with 3 three signs from the non-CI group. The cut-off values were 7.3 2 cm for NPC, -0.23 to 1.00 for Sheard's criterion, and -4.00 to -2.33 for Percival's criterion. Our results suggest that the use of Sheard's criterion with NPC shows high performance for screening of CI.

Introduction

Non-strabismic binocular vision anomalies consist mostly of accommodative and vergence disorders. Vergence disorders are classified into convergence insufficiency (CI), convergence excess, divergence insufficiency, divergence excess, basic esophoria, basic exophoria, and fusional vergence dysfunctions based on phoria and the accommodative convergence to accommodation ratio (AC/A) [1, 2]. CI is a ~~common~~ dysfunction of binocular vision ~~disorder~~ that is associated with various symptoms, including blurred vision, diplopia, and discomfort while performing near work [3]. The signs of CI include exophoria that is greater at near than at distance, reduced near point of convergence (NPC), reduced positive fusional vergence (PFV), low AC/A, and deficiencies in negative relative accommodation (NRA) [1]. However, cases with CI can be simple or complex aspects because not all patients with CI have all ~~of~~ these symptoms and signs [1, 4]. The reported prevalence of CI varies widely from 1.0% to 33% [1, 5-7]. These wide variations can be attributed to the differences in patient age, sample populations, and diagnostic criteria used in the previous studies [8]. Therefore, it is important to evaluate the ability of clinical diagnostic tests to discriminate between CI and other disorders, since the clinical criteria used for screening CI have been different across several studies.

Receiver operating characteristic (ROC) curve analysis is useful for evaluating the quality or performance of diagnostic tests. In a very recent study [9], ROC curve analysis was used to ~~evaluate the ability~~assess the performance of PFV, NPC, AC/A, accommodative amplitude (AA), binocular accommodative facility (BAF), ratio of PFV over the phoria (PFV/phoria), and the convergence insufficiency symptom survey (CISS) to discriminate between school-age children with and without CI. The results of that study indicated that the NPC break performed best in identifying

children with CI. However, these results cannot be extended to adults since that study was limited to patients aged 9 to 18 years and because of the limited validity of the Insufficiency Symptom Survey (CISS) used for children [10]. A previous study used ROC analysis to investigate the diagnostic validity of the clinical signs of CI in participants aged 19 to 35 years with either symptomatic large exophoria and normal heterophoria, or low visual discomfort at near [11]; it reported that In a study comparing large exophoria and normal phoria among participants aged 19 to 35 years [11], NPC and BAF tests showed the best diagnostic accuracy for discriminating between the two groups. In another ROC curve analysis of only BAF and vergence facility (VF) in university students with symptomatic CI and normal participants classified using the CISS [12], BAF showed greater accuracy than VF for diagnosis of CI. All these previous studies have evaluated the validity of diagnostic tests between symptomatic patients with CI and normal participants. However, in addition to cases of independent CI, CI can present with signs associated with other vergence disorders or accommodative disorders in clinical diagnoses of non-strabismic binocular vision anomalies [13]. The symptoms of CI are common in adults, but they often do not appear [1, 5]. Thus, a careful differential diagnosis is required to identify this condition.

The first consideration for screening CI in the presence of non-strabismic binocular vision anomalies is to distinguish it from accommodative dysfunction through the analysis of the values or signs measured by each test for accommodative and vergence functions used in the evaluation of binocular vision. The second approach involves identification of CI-related signs. The final stage approach requires identification of a close correlation between symptoms and clinical signs. In the final approach, Hhowever, the symptoms in the final approach may not be related to

clinical signs, especially in adults. Many symptomatic CI disorders are related to the presence of defects in two or more areas of binocular vision function [13, 14].

Our study was limited to young adults and an approach focused on signs and modified signs such as Sheard’s and Percival criterion, in addition to fusional vergence related to ability to maintain single binocular vision. The purpose of this study was to evaluate the accommodative and vergence ability for university students who visited for primary eye care due to ocular discomfort and to analyze the diagnostic ability of each test for screening CI by performing ROC curve analysis, including assessments of sensitivity, specificity, cut-off values, and likelihood ratio.

Materials and ~~M~~methods

Subjects

The participants were 184 university students (age~~,~~ = 18-28 years; mean age~~,~~ = 22.23 ± 2.26 years) who underwent an eye examination due to ocular discomfort. Participating students voluntarily visited a university eye clinic center for primary eye care due to blurred vision, eyestrain, and visual discomfort. This study was approved by the Kangwon ~~n~~National ~~u~~University ~~i~~Institutional ~~r~~Review ~~b~~Board (KWNUIRB-2019-02-001-002), with waiver of the informed consent for the retrospective collection of clinical data, and adhered to the tenets of the Declaration of Helsinki. Participants had not previously undergone any vison therapy or eye exercise treatment. We excluded patients with ocular diseases such as glaucoma, cataract, and retinal disease and those with a history of prior surgery, which was determined by history-taking [1]. The criteria for inclusion into study also were the absence of amblyopia and strabismus.

General procedure

Before refraction assessments, all participants underwent a case evaluation to obtain information about the ocular discomfort, followed by ~~an~~ ocular motility testing and a cover-uncover test to rule out strabismus. Refraction and binocular vision tests were performed using a phoropter (VT-SE; Topcon ~~c~~Corporation, Tokyo, Japan) and visual charts (ACP-8; Topcon ~~c~~Corporation, Tokyo, Japan) at distance (6 m) ~~or~~ and near (40 cm). All measurements were taken in a general clinical room by the same examiner, who performed all tests within approximately 30 minutes, using the same methodology.

Assessment of binocular vison function

Following pre-refraction and the best-corrected refraction of ≥20/20 visual acuity, the NPC was measured in free space by using an isolated 20/50 target on a Gulden fixation stick to evaluate the subjects' ability to converge the eyes while retaining binocular single vision [15]. The measured value was determined by the distance (cm) from the subject's eye or spectacle plane at which the subject reported double vision when the target was moved at a rate of approximately 5 cm/s toward the subject ~~at the eye level and between the both eyes~~between both eyes and at eye level. The normative value is 5 ± 2.5 cm for break point of NPC.

Phoria was measured by using the von Graefe technique [16] to assess the direction and amplitude of eye alignment, which indicated latent misalignment of the eyes. Horizontal phoria was measured with a measuring prism of 12 Δ (prism) base in (BI) and a dissociating prism of 6 Δ base up (BU) before one eye and the fellow eye, respectively. The measured value was the amount and direction of the prism when the

measuring prism of 12 Δ BI was reduced at a rate of approximately 2 Δ/s until the subject reported vertical alignment of the lower and upper 20/30 letter target separated by the two prisms. After measurement of phoria at distance, near phoria was measured. Negative values represent exophoria, whereas positive values represent esophoria. The normative values for distance phoria and near phoria are 1exo ± 1 Δ and 3exo ± 3 Δ, respectively.

Binocular AA is the maximum amount of accommodation under binocular conditions, which represents the visual function required for maintaining a clear image [17]. AA was measured by the push-up method by using an accommodative convergence rule (GR50, Bernell, USA) and a near target (near visual acuity, 20/30). The near point of accommodation was determined by assessing the distance from the subject's eye or spectacle plane to the target at which the subject reported when the target first became blurredthe subject reported blurred vision when the target was movedwhile moving at a rate of approximately 2 cm/s closer towards the subject's eyes. The amount of AA was expressed in diopter (D) by the inverse of the near point of accommodation (m). The normative value for mean AA is 18.5 − (1/3) age.

Fusional vergences were measured by using the rotary prism on the phoropter and a 20/30 letter target to evaluate the subject's ability to use horizontal vergence to maintain binocular vision. First, negative fusional vergences (NFVs) at distance were measured by using the BI prism [1817]. The prism power was increased in front of both eyes at the rate of 1–2 Δ/sec until subjects reported a blurred image (blur point) and then a double image (break point), and then decreased to a find a recovery point until the subject reported a single image. PFVs at distance were measured by base out (BO) prism with the same method as NFVs. Fusional vergences at near were measured after fusional vergence tests at distance [17]. If no blur value in fusional

vergence was reported, the break value was used in the analysis. The normative values are 9 ± 2 Δ for bur, 19 ± 4 Δ for break, and 10 ± 2 Δ for recovery of PFV at distance; 7 ± 2 Δ for break, and 4 ± 1 Δ for recovery of NFV at distance; 17 ± 3 Δ for bur, 21 ± 3 Δ for break, and 11 ± 4 Δ for recovery of PFV at near; 13 ± 2 Δ for bur, 21 ± 2 Δ for break, and 13 ± 3 Δ for recovery of NFV at near.

Gradient AC/A ratio (AC/A) was determined as the difference in phoria between before and after the addition of + 1.00 D at near divided by 1.00 D. Calculated AC/A was determined as the sum of inter-pupillary distance ~~in cm~~(cm), measured by PD meter and the difference in phoria between near and distance divided by 2.50 D [~~19~~17, 18]. The normative value for AC/A is 4 ± 1 Δ.

Relative accommodations were measured to examine the subject's ability to increase and decrease accommodation under binocular vision when the convergence demand was constant [~~20~~19]. Negative relative accommodation (NRA) was measured first, adding plus power over the refraction at the rate of 0.25 D/2 s. The measured values were the amount of plus power added until the subject reported the first maintained blur. Positive relative accommodation (PRA) was measured as the minus power added over the refraction until the sustained blur. The normative values for NRA and PRA are + 2.00 ± 0.25 D and −2.37 ± 0.62 D, respectively.

BAF was measured at 40 cm by using a ± 2.00 D binocular flipper lens to evaluate the ability of the accommodative response at near [~~21~~20]. If the subject reported clear vision for a 20/30 letter target when +2.00 D was placed in both eyes, the lens was flipped to place -2.00 D in both eyes. When the subject ~~again~~ reported clear vision again, this indicated one cycle. Measured values were in terms of the number of cycles per minute (cpm). The normative value for BAF is > 13 cpm.

Vergence facility (VF) was measured by using a prism flipper (3 Δ BI + 12 Δ

~~BU~~BO) to evaluate the ability of the fusional vergence at near (40 cm) [~~22~~21]. If the subject reported ~~clear~~ single vision with a 20/30 letter target when 12 Δ ~~BU~~ BO was placed in both eyes, the prism was flipped to place the 3 Δ BI in both eyes. When the subject ~~again~~ reported clear vision again, this indicated one cycle. Measured values were in terms of the number of cycles per minute (cpm). The normative value for VF is > 12 cpm.

The relation of fusional vergence to phoria was analyzed by using Sheard's [~~23~~22] and Percival's criteria [~~24~~23]. Sheard's criterion aimed to determine whether the blur point of fusional vergence is at least twice the phoria. The values for Sheard's criterion were calculated as 2/3 the phoria minus 1/3 the fusional vergence. Vergence anomalies were considered to exist when the subjects failed to meet this criterion (value of Sheard's criterion > 0). Percival's criterion was that the orthophoria point of subject should be operating in the middle-third of the binocular vergence range. The values for Percival's criterion were determined by calculating the value 1/3 greater of two fusional vergences minus 2/3 lesser of the two fusional vergences. If the obtained value was positive, vergence anomalies were considered to be present.

All data were compared with Morgan's expected findings [~~25~~17, 24], and the main characteristic signs were identified to diagnose accommodative and vergence anomalies. The diagnosis of accommodative and vergence anomalies was classified by signs based on Table 1, which ~~were modified from the study by~~referred to Scheiman and Wick's study [17] and compared with the expected criteria for each test [~~25~~24]. Subjects groups were classified into three groups according to Table 1: All CI including CI with two signs and CI with an accommodative dysfunction, other binocular vision anomaly (BVA) except CI, and normal binocular vision (NBV). All CI were also classified into CI with three signs (CI3), CI with two signs and an

accommodative dysfunction (CI3AD), and CI with two signs (CI2).

Table 1. Diagnostic criteria for non-strabismic binocular vision anomalies.

Convergence insufficiency (CI)
Presence of sign~~s~~ 1 and sign 2 or 3
1. Exophoria at near ≥ 4 Δ than at distance
2. PFV at near < 14 Δ for blur or <18 Δ for break
3. NPC ≥ 7.5 cm

Divergence insufficiency (DI)
Presence of sign~~s~~ 1 and sign 2 or 3
1. Esophoria at distance ≥ 4 Δ than at near
2. NFV at distance < 5 Δ for break
3. Gradient or calculated AC/A ratio < 3/1

Convergence excess (CE)
Presence of sign~~s~~ 1 and sign 2 or 3
1. Esophoria at near ≥ 4 Δ than at distance
2. NFV at near < 11 Δ for blur or <19 Δ for break
3. Gradient or calculated AC/A ratio > 5/1

Divergence excess (DE)
Presence of sign~~s~~ 1 and sign 2 or 3
1. Exophoria at distance ≥ 4 Δ than at near
2. PFV at distance < 7 Δ for blur or <15 Δ for break
3. Gradient or calculated AC/A ratio > 5/1

Basic exophoria (BX)
Presence of sign~~s~~ 1 and sign 2 or 3
1. Exophoria < 4 Δ difference between distance and near
2. PFV at distance < 7 Δ for blur or <15 Δ for break, and PFV at near < 14 Δ for blur or <18 Δ for break
3. Gradient or calculated AC/A ratio 3–5/1

Basic esophoria (BE)
Presence of sign~~s~~ 1 and sign 2 or 3
1. Esophoria < 4 Δ difference between distance and near
2. NFV at distance < 5 Δ for break & NFV at near < 11 Δ for blur or <19 Δ for break
3. Gradient or calculated AC/A ratio 3–5/1

Fusional vergence dysfunctions (FVD)
Presence of sign~~s~~ 1 and sign 2 or 3
1. Normal phoria at distance (1 exo ± 1Δ) and near (3 exo ± 3Δ)
2. Reduced (low) PFV and NFV at distance and near
3. Gradient or calculated AC/A ratio 3–5/1

Accommodative insufficiency (AI)
Presence of sign~~s~~ 1 and sign 2 or 3
1. Reduced binocular AA for minimum amplitude = 15.0 - 0.25 × age
2. Low PRA < 1.75 D
3. BAF~~inocular accommodative facility~~ < 13 cpm

Accommodative excess (AE)
Presence of signs 1 and 2
1. Low NRA < 1.75 D
2. BAF < 13 cpm

Accommodative infacility
Presence of signs 1 and 2
1. Low NRA & PRA < 1.75 D
2. BAF < 13 cpm

D: diopter, Δ: prism diopter, cpm: cycles per minute, AA: accommodative amplitude, NPC: near point of convergence, AC/A: accommodative convergence/accommodation, NFV: negative fusional vergence, PFV: positive fusional vergence, NRA: negative relative accommodation, PRA: positive relative accommodation, BAF: binocular accommodative facility.

Data analysis

Data were collected and analyzed using IBM SPSS Statistics version 19 (IBM Corp., USA). One-way ANOVA with a Bonferroni's *post-hoc* test comparing the three groups (all CI, BVA, and NBV) was performed using mean and standard deviation values. A p-value of ~~≤~~ < 0.05 was considered significant. ROC curve analysis was

performed by plotting sensitivity on the y axis as a function of 1 – specificity on the x axis to analyze the diagnostic ability of each test (AA, NPC, AC/A, NFV, PFV, NRA, PRA, BAF, VF, Sheard's and Percival's criterion) in screening CI. Sensitivity refers to the probability that a test will indicate CI when CI is present, and specificity refers to the probability that a test will indicate the absence of CI when CI is not present. The area under the curve (AUC) in the ROC curve analysis indicates the discriminative ability to distinguish between subjects with and without CI. The cut-off value for each test was defined as the coordinate that had the maximized sum of sensitivity and specificity. The cut-off was also identified for each test with the largest AUC which was significantly greater than 0.50. The likelihood ratio indicates the degree of increase or decrease in the probability of the CI if the test yields positive or negative findings.

Results

In an assessment of the refractive errors in the participants, of the 368 eyes, 258 (70.1%) showed myopia (spherical equivalent [SE] = -3.31 ± 2.16 D), 19 (5.2%) showed hyperopia (SE = +0.80 ± 0.51 D), and 91 (24.7%) showed emmetropia. Myopia was present in 125 participants (67.9%), hyperopia in six participants (3.3%), emmetropia in 42 participants (22.8%), mixed anisometropia in four participants (2.2%), simple myopic anisometropia in four participants (2.2%), and simple hyperopic anisometropia in three participants (1.6%).

The means and standard deviations for each test of binocular vision function after wearing refractive correction are shown in Table 2. Subjects were divided into three groups: all CI (n = 39); binocular vision anomalies (BVAs; n=49) except CI; and normal binocular vision (NBV; n=96). The one-way ANOVA with Bonferroni's *post-*

hoc test showed significant intergroup differences in ~~AA~~ NPC, near phoria, and calculated AC/A; significant differences between all CI and other BVA, and other BVA and NBV in distance phoria and distance~~near~~ NFV; and significant differences between all CI and NBV in gradient AC/A, PFV blur and recovery at distance, near PFV, NRA, PRA, BAF, and VF.

Table 2. Comparison of mean and standard deviation values for the measures of binocular function.

Test	All subjects (n = 184)	Subject groups			p-value (*post-hoc*)
		a. All CI (n = 39)	b. Other BVA (n = 49)	c. NBV (n = 96)	
AA (D)	11.46 ± 2.48	~~1.00~~ 10.90 ± 1.75	11.24 ± 2.83	11.79 ± 2.51	0.130
NPC (cm)	7.29 ± 1.93	9.21 ±2.07	7.24 ± 1.85	6.53 ± 1.27	< 0.001* (a>b>c)
Phoria at distance (Δ)†	-2.28 ± 2.91	-3.04 ± 2.10	-0.88 ± 4.04	-2.69 ± 2.20	< 0.001* (a, c>b)
Phoria at near (Δ)†	-6.34 ± 5.63	-10.37 ± 2.84	-1.71 ± 5.93	-7.10 ± 4.63	< 0.001* (a>c>b)
Calculated AC/A (Δ/D)	4.82 ± 1.82	3.55 ± 1.33	6.16 ± 2.06	4.66 ± 1.38	< 0.001* (b>c>a)
Gradient AC/A (Δ/D)	3.14 ± 1.62	2.40 ± 1.42	3.54 ± 1.73	3.23 ± 1.55	< 0.003* (b, c>a)
NFV break at distance (Δ)	9.11 ± 3.99	9.72 ± 3.80	7.78 ± 3.64	9.54 ± 4.12	0.023* (a, c>b)
NFV recovery at distance (Δ)	5.28 ± 2.91	6.15 ± 3.10	4.25 ± 2.45	5.46 ± 2.92	0.006* (a, c>b)
PFV blur at distance (Δ)	10.08 ± 4.51	8.69 ± 3.18	9.02 ± 3.95	11.19 ± 4.97	0.002* (c>a, b)
PFV break at distance (Δ)	16.16 ± 7.84	14.62 ± 5.95	14.04 ± 7.40	17.87 ± 8.39	0.008* (c>b)
PFV recovery at distance (Δ)	9.76 ± 6.57	8.03 ± 4.68	8.55 ± 5.87	11.08 ± 7.30	0.015* (c>a)
NFV blur at near (Δ)	13.47 ± 5.19	14.10 ± 5.00	12.10 ± 5.72	13.91 ± 4.91	0.097
NFV break at near (Δ)	19.92 ± 6.55	21.00 ± 5.67	18.25 ± 7.84	20.33 ± 6.05	0.097
NFV recovery at near (Δ)	13.51 ± 5.85	14.03 ± 4.99	12.47 ± 6.80	13.82 ± 5.65	0.348
PFV blur at near (Δ)	15.52 ± 6.23	13.41 ± 6.41	13.41 ± 6.36	17.45 ± 5.47	< 0.001* (c>a, b)
PFV break at near (Δ)	21.15 ± 8.48	17.92 ± 8.10	19.08 ± 8.63	23.51 ± 7.90	< 0.001* (c>a, b)
PFV recovery at near (Δ)	13.39 ± 8.70	11.10 ± 8.71	11.43 ± 7.61	15.32 ± 8.85	0.006* (c>a, b)
NRA (D)	2.15 ± 0.63	1.87 ± 0.65	1.92 ± 0.65	2.39 ± 0.50	< 0.001* (c>a, b)
PRA (D)	-2.77 ± 1.15	-2.76 ± 1.26	-2.40 ± 1.11	-2.98 ± 1.10	0.017* (c>b)
BAF (cpm)	12.47 ± 5.64	10.74 ± 5.61	12.12 ± 6.63	13.35 ± 4.95	0.044* (c>a)
VF (cpm)	13.27 ± 3.93	10.59 ± 4.09	13.61 ± 4.04	14.19 ± 3.31	< 0.001* (c>a)
Sheard's criterion at distance	-1.22 ± 2.00	-0.87 ± 1.80	-0.30 ± 2.15	-1.83 ± 1.80	< 0.001* (a, b>c)
Sheard's criterion at near	-0.15 ± 3.25	2.44 ± 2.75	-0.69 ± 2.98	-0.92 ± 3.05	< 0.001* (a>b, c)
Percival's criterion at distance	-1.11 ± 1.69	-1.27 ± 1.39	-0.54 ± 1.60	-1.32 ± 1.80	0.023 (b>c)
Percival's criterion at near	-1.77 ± 2.34	-1.11 ± 2.32	-1.23 ± 2.34	-2.32 ± 2.23	0.004 (a, b>c)

CI: convergence insufficiency, BVA: binocular vision anomaly, NBV: normal binocular vision, D: diopter, Δ: prism diopter, cpm: cycles per minute, AA: accommodative amplitude, NPC: near point of convergence, AC/A: accommodative convergence/accommodation, NFV: negative fusional vergence, PFV: positive fusional vergence, NRA: negative relative accommodation, PRA: positive relative accommodation, BAF: binocular accommodative facility, VF: vergence facility. $^*p < 0.05$ indicates statistically significant differences among groups in one-way ANOVA followed by Bonferroni's *post-hoc* test. †Minus and plus signs in phoria indicate exophoria and esophoria, respectively.

Table 3 shows the prevalence of ~~non-strabismic~~ binocular vision anomalies diagnosed according to Table 1 based on data for each test prior to the ROC curve analysis. It also shows the characteristics of the subjects enrolled in CI screening assessments. Of these 184 young adults, 96 (52.2%) were classified as ~~the normal group~~NBV and 34 (18.5%) were identified as having ~~2 signs of CI~~ CI2. CI with

accommodative disorders (CI + AI and CI + AE) was observed in five (2.7%) participants, while the prevalence of CI varied from 10.3% (19 participants with CI3) to 21.2% (39 participants with CI2AD) depending on the number of signs and the presence of associated accommodative disorders. When participants with BVAs other than CI were referred to as non-CI, the distribution between CI and non-CI was different according to the classification criteria.

Table 3. Prevalence of ~~non-strabismic~~ binocular vision anomalies.

Dysfunction	n	%	CI subjects associated with signs and AD
CI	34	18.5	
DI	11	6.0	
CE	4	2.2	
DE	2	1.1	Classified CI vs. non-CI group[‡]
BX	9	4.9	
BE	4	2.2	CI + 3 signs (CI3): 19 (10.3%) vs. ~~179~~ 165 (89.7%)
AI	7	3.8	
AE	2	1.1	CI + 3 signs + AD (CI3AD): 23 (12.5%) vs. 161 (87.5%)
CI + AI	4	2.2	
CI + AE	1	0.5	CI + 2 signs (CI2): 34 (18.5%) vs. 150 (81.5%)
CE + AE	3	1.6	
BX + AI	1	0.5	CI + 2 signs + AD (CI2AD): 39 (21.2%) vs. 145 (78.8%)
BE + AI	1	0.5	
Indefinite	5	2.7	
NBV	96	52.2	
Total	184	100	

Abbreviations are the same as those in the notes for Table 1. Signs are sign 1, 2, 3 for CI in Table 1. AD: accommodative dysfunction, CI3: CI with 3 signs, CI3AD: CI with 3 signs + CI with an accommodative dysfunction, CI2: CI with 2 signs, CI2AD: CI with 2 signs + CI with an accommodative dysfunction. [†]Non-CI group is a group excluding CI group.

ROC curve analysis was performed for the tests shown in Table 2. The results of the AUC for NPC, Sheard's and Percival's criterion with $p < 0.05$ and AUC > 0.5 in 95% confidence interval ranges, and PFV, NFV, and AC/A including the diagnostic criteria in Table 1~~The AUC findings (p < 0.05 and AUC > 0.5 in 95% confidence interval ranges) from ROC curve analyses for each CI test in various conditions~~ are shown in Table 4. Fig 1 shows ROC curves for NPC, Sheard's and Percival's criterion~~each test~~ with statistically significant differences when comparing the AUC

to the value 0.5. Among the AUCs for all diagnostic tests included in Table 1, the values were significantly greater than 0.5 for only the NPC and NFV at distance, and the AUCs for diagnostic tests excluded in Table 1 were also significantly greater than 0.5 for Sheard's and Percival's criteria. AUCs were greater for NBV than for non-CI, greater for excluding than for including CI associated with AD, and greater for CI with ~~3~~ three signs than for CI with ~~2~~ two signs.

Table 4. Results for the area under the curve (AUC) in ROC curve analysis among CI, non-CI, and NBV groups.

~~Screening for various CI conditions~~ Ability to discriminate CI		NPC	Sheard†	Percival†	NFV	PFV^{ns}	AC/A^{ns}
~~CI3~~	From CI3 + NBV	0.920	0.912	0.717	(0.627) rec	< 0.2	< 0.3
	From CI3 + non-CI	0.842	0.905	0.672	(0.654) rec	< 0.2	< 0.3
~~CI3AD~~	From CI3AD + NBV	0.920	0.905	0.702	0.577^{ns} brk	< 0.2	< 0.3
	From CI3AD + non-CI	0.853	0.900	0.657	0.601^{ns} brk	< 0.2	< 0.3
~~CI2~~	From CI2 + NBV	0.914	0.793	0.652	(0.614) rec	< 0.4	< 0.3
	From CI2 + non-CI	0.866	0.773	0.608	(0.656) rec	< 0.5	< 0.3
~~CI2AD~~	From CI2AD + NBV	0.913	0.804	0.642	(0.566)^{ns} rec	< 0.4	< 0.4
	From CI2AD + non-CI	0.876	0.795	0.598	(0.603) rec	< 0.4	< 0.4

Abbreviations are the same as those in the notes for Tables 1, 2, and 3. †Criterion for near, brk: break point, rec: recovery point, ^{ns}: not significant, ( ): value for distance.

Fig 1. ROC curves for NPC and Sheard's and Percival's criterion according to the combined CI groups.

Blue ~~S~~solid line: NPC, red dotted line: Sheard's criterion for near, dashed line: Percival's criterion for near, diagonal line: no discrimination (AUC = 0.5).

Table 5 shows the sensitivity, specificity, positive likelihood ratio (LR+), and negative likelihood ratio (LR-) for each test by using cut-offs obtained with ROC curves. The NPC cut-offs were >7.~~3~~ 2 cm for all classified CIs combined with the

NBV and non-CI groups. The Sheard's criterion cut-off of >1.00 for CI with ~~3~~ three signs (CI3) was higher than the cut-off of >-0.23 for CI with ~~2~~ two signs (CI2), and showed higher sensitivity and specificity than CI2. The Percival criterion cut-off of >-4.00 for CI2 was lower than the cut-off of >-2.33 for CI3. Sensitivity, specificity, and LR+ were higher in the order of NPC, Sheard's, and Percival's criterion, but LR- showed the opposite trend.

Table 5. Diagnostic validity of NPC and Sheard's and Percival's criterion by using cut-offs derived from ROC curve analysis

Screening for combined CI groups			Cut-off	Sensitivity	Specificity	LR+	LR-
CI3	+ NBV	NPC	>7.20	1.00	0.84	6.40	0.00
		Sheard	>0.33	1.00	0.74	3.84	0.00
		Percival	>-2.33	0.84	0.56	1.92	0.28
	+ non-CI	NPC	>7.20	1.00	0.68	3.11	0.00
		Sheard	>0.33	1.00	0.67	3.06	0.00
		Percival	>-2.33	0.84	0.47	1.60	0.33
CI3AD	+ NBV	NPC	>7.20	1.00	0.84	6.40	0.00
		Sheard	>0.33	1.00	0.74	3.84	0.00
		Percival	>-2.33	0.83	0.56	1.89	0.31
	+ non-CI	NPC	>7.20	1.00	0.70	3.29	0.00
		Sheard	>1.00	0.96	0.74	3.67	0.06
		Percival	>-2.33	0.83	0.48	1.58	0.36
CI2	+ NBV	NPC	>7.20	1.00	0.84	6.40	0.00
		Sheard	>-0.23	0.82	0.69	2.64	0.26
		Percival	>-2.33	0.71	0.56	1.61	0.52
	+ non-CI	NPC	>7.20	1.00	0.75	3.95	0.00
		Sheard	>-0.23	0.82	0.63	2.25	0.28
		Percival	>-4.00	0.97	0.24	1.28	0.12
CI2AD	+ NBV	NPC	>7.20	1.00	0.84	6.40	0.00
		Sheard	>-0.23	0.85	0.69	2.71	0.22
		Percival	>-4.00	0.97	0.28	1.36	0.09
	+ non-CI	NPC	>7.20	1.00	0.77	4.39	0.00
		Sheard	>-0.23	0.85	0.66	2.45	0.23
		Percival	>-4.00	0.97	0.25	1.30	0.10

Abbreviations are the same as those in the notes for Tables 1, 2, and 3. LR+: positive likelihood ratio, LR-: negative likelihood ratio.

Discussion

In this study, with ROC curve analysis of signs, the tests that showed a significant discriminative ability between CI and non-CI or CI and NBV among young adult~~s~~ university students were those based on the Sheard’s and Percival’s criteria for near vision, while NPC assessment was the best diagnostic test for identifying CI. For CI screening between CI3 (CI with ~~3~~ three signs) and non-CI, Sheard’s criterion was a better diagnostic parameter than NPC. The distribution of CI, non-CI, and NBV according to diagnostic criteria such as population and the signs of CI influenced the validity of each test in the ROC curve analysis.

The prevalence of CI in this study ranged between 10.3% and 21.2%, higher than rates of 1.5% to 10.8% reported in previous studies ~~in~~ of the general population–university students [8, ~~26~~25, ~~27~~26]. These variations in the prevalence of CI can be attributed to differences in methodological aspects, including instrumentation and techniques, classification criteria, and the number of diagnostic signs; the types of populations studied (clinical/non-clinical); data analysis methods; and participant factors including age as well as refractive errors [~~28~~27]. Our study was performed in university students who underwent primary eye care due to ocular discomfort, and CI was classified based on ~~the basis of~~ signs. The CI screening ability of each test was also evaluated in ~~conditions such as~~ NBV and non-CI conditions. The variation in CI prevalence in this study appears to be associated with differences in signs, classification criteria, and population. In our study, the prevalence of myopia was high (70.1%). CI has been reported to show a significant association with myopia [~~29~~28], whereas CI and refractive errors were not significantly associated [~~30~~29]. Although two previous studies showed different results, the high prevalence of CI in this study may be partially explained by the association with myopia. CI may present as types associated with accommodative dysfunction such as CI plus ~~accommodative~~

~~insufficiency (~~AI~~)~~ [~~31~~30] or CI plus accommodative excess (AE) [~~20~~19]. CI combined with accommodative functions can also exist because the vergence and accommodative systems are linked [~~32~~31]. ~~Therefore,~~ CI should also be distinguished from ~~these~~ conditions such as normal vision, diverse vergence and accommodative disorders, and other binocular anomalies. Our study factored these conditions to investigate the discriminative ability of each test for CI screening by performing ROC curve analysis under these conditions.

In comparisons of the means and standard deviations for each test, assessments based on NPC, AC/A, PFV blur/break/recovery at near, NRA, BAF, and VF showed significant differences between all-CI and NBV groups, and those based on NPC and AC/A showed significant differences between all-CI and other BVA groups. Tests with a significant difference were noted less frequently in comparisons with other BVA than in comparisons with NBV, as shown in Table 2. In Cacho-Martínez et al.'s [11] study comparing large phoria (>6 Δ) and normal phoria, significant differences were found on 5 tests such as NPC, PFV blur at near, NRA, BAF, and VF. However, in our study comparing all-CI and other BVA groups, significant differences were found on 2 tests of NPC and AC/A. This finding indicates that it is difficult to distinguish CI from other BVA. Since patients with CI ~~have to~~must be distinguished from subjects with various other clinical conditions, the efficiency of CI screening depends on the performance of the diagnostic test in discriminating CI from abnormal groups combined with other binocular anomalies rather than normal groups. Tests for the diagnosis of CI were based on the ratio of positive fusional vergence to phoria [9] and Sheard's criterion in other studies [~~23~~22, ~~33~~32], and these tests was limited to school-age children. However, Percival's criterion as well as Sheard's criterion was applied to adults in this study. There are significant differences between the all-CI and

NBV groups for Sheard's criterion at distance/near and Percival's criterion at near and between the all-CI and other-BVA groups for Sheard's criterion at near. These results indicate that Sheard's criterion could be used in tests to distinguish CI from other BVA and NBV, and Percival's criterion could be used to distinguish CI from NBV. However, Sheard's criterion is a useful tool for screening CI with exophoria associated with near tasks because the signs of CI include exophoria more than 6 Δ at near and normal phoria of 0–6 Δ exophoria at distance [~~25~~17], and previous studies [~~34~~33, ~~35~~34] have suggested that Sheard's and Percival's criteria are the most effective with exophoria and esophoria, respectively.

The main finding in this study was that NPC can distinguish individuals with CI signs or CI signs associated with AD, namely, CI2AD and CI3AD from the NBV and non-CI groups. In ROC curve analysis, the AUC of 0.842–0.920 obtained using the NPC test represents an excellent discriminative ability for CI screening. Although the test parameters such as subjects and diagnostic and classification criteria were not consistent with other studies [25, 26], our result is consistent with outcomes that suggest that NPC is the best in identifying children with CI [9] and subjects with large near exophoria and moderate to severe symptoms [11]. The discriminative ability in CI groups (CI3, CI3AD, CI2, CI2AD) combined with NBV is higher than that in CI groups combined with non-CI. The NPC test showed a cut-off value of >7.2 cm in comparison with previously reported values of 7 cm [11] or 7.5 cm [~~36~~35, ~~37~~36] and sensitivity of 1.00 in all groups, and specificity and positive likelihood ratio (LR+) were higher in the combined group of CI and NBV than in the combined group of CI and non-CI. A high LR+ indicates a high ratio of the probability of the true presence of CI to the probability of false presence of CI in the NPC test. A low LR- indicates a low ratio of the probability of false absence of CI to the probability of true absence of

CI in the NPC test. A negative likelihood ratio (LR-) of zero in all classified CIs indicates a decreased probability that the NPC test is negative.

The ROC curve analysis in this study showed that Sheard's and Percival's criteria have potential for use as tools for CI screening. Sheard's criterion is particularly useful for CI screening from non-CI than from NBV. In addition, Sheard's criterion can be a better tool than NPC in cases of CI with ~~3~~ three signs (CI3, CI3AD). The previous studies [9, 11] did not provide cut-off values, sensitivity, specificity, AUC, and other data from ROC curve analysis for Sheard's and Percival's criterion to screen CI. The AUC of 0.773– 0.912 obtained using Sheard's criterion in our study represents an acceptable discriminative ability for CI screening, and the AUC reduced with decreasing signs. Sheard's criterion in this study has positive cut-offs values (failed to normal binocular vision or needed prism) in the CI3 and cut-offs of > -0.23 (approximately cut-offs > zero) in the CI2. Sheard's criterion could diagnose all-CI. The sensitivity of the combined CI3 is higher than that of the combined CI2, and the specificity is lower than sensitivity. Although the LR+ of 2.25–3.84 was lower than the corresponding value for the NPC assessment as a positive test result indicating the presence of CI, and the LR- of 0.22–0.28 was higher than the corresponding value for the NPC assessment as a negative result test indicating the absence of CI, from another perspective, this criterion is a valid tool for discriminating CI with ~~3~~ three signs (CI3, CI3AD) from non-CI because the AUC of Sheard's criterion was greater than that in NPC and the LR- of zero in CI3 and ~~CI3 with AD (~~CI3AD~~)~~ was equal to that for NPC.

Percival's criterion showed cut-off values of <0 (negative value; meet criterion or not needed prism) in all groups. Although Percival's criterion showed cut-off values for screening CI, the ROC curve analysis values indicate that it is a valid tool for CI

screening when considering AUC ≥ 0.598, sensitivity ≥ 0.71, specificity of 0.24 – 0.56, LR+ ≥ 1.28, and LR- ≤ 0.36. Values of for CI screening showed a mismatch between Percival's criterion (the amount of prism required or a positive cut-off value) and the results of ROC curve analysis (a negative cut-off value) but an approximate match between Sheard's criterion (the amount of prism required or a positive value) and the results of ROC curve analysis (cut-off value of -0.23 close to a positive). These differences could have occurred because Sheard's criterion works best for exophoric conditions such as CI and Percival's criterion tends to work best for near esophoric conditions such as convergence excess [2423, 3837]. However, Percival's criterion showed a lower discriminative ability than Sheard's criterion for CI screening. In ROC curve analysis, Percival's criterion showed lower AUC and LR+ than Sheard's criterion. Percival's criterion also showed lower sensitivity than Sheard's criterion, except for CI2 combined with non-CI (CI2 + non-CI) and CI2 combined with AD (CI2AD + NBV, CI2AD + non-CI). The LR- of Percival's criterion was higher than that of Sheard's criterion except for CI2 combined with non-CI and combined CI2AD. Although Percival's criterion could be a useful tool for CI screening, this criterion needs to be modified to show better diagnostic accuracy for binocular vision disorders. The ROC curve analysis for each test except the above showed no statistically significant differences for CI screening, but significant differences were noted for BAF in other studies [9, 12]. On the other hand, a high PRA was shown to be the most sensitive sign for CI combined with accommodative excess [2019], and Gall at al. [2221] found that VF can differentiate symptomatic from asymptomatic patients (not assessed in this study). However, BAF, PRA, and the VF test were not shown to be useful in discriminating CI from the normal or non-CI groups in the results of our study. Thus, factors related to the classification criteria for

binocular vision disorders and subject characteristics such as age and population might lead to a different result.

Although this study was conducted with participants reporting ocular discomfort, one limitation of this study is the lack of evaluation about subjective symptoms since the analysis was based on objective clinical signs. In the previous studies, some adults with CI signs were asymptomatic [~~39~~38] and there was no further association between the severity of the clinical signs and symptoms in children aged 9 to 17 years [4]. On the other hand, other studies have shown an association between signs and symptoms [9, ~~40~~39]. These results cannot extend to young adults. Therefore, the relationship between signs and symptoms in university students needs further study.

In summary, this study shows that Sheard's and Percival's criteria are useful tools to discriminate CI in young adults although the NPC test has diagnostic validity for screening subjects with CI signs from not only NBV but also non-CI with AD and other binocular vision disorders. On the other hand, with respect to the AUCs of the ROC curve analysis for CI screening in cases of CI with three signs, Sheard's criteria were significantly greater than NPC and Percival's criteria. In addition, this study suggests that Percival's criterion such as "orthophoria point should be in the middle third of the vergence range" needs to be revised due to showing acceptable sensitivity and specificity for CI screening at a negative cut-off value within the range to meet this criterion.

Supporting Information

S1 File. All relevant raw data.

(XLSX)

Acknowledgements

Not applicable.

References

1. Cooper JS, Burns CR, Cotter SA, Daum KM, Griffin JR, Scheiman MM. Optometric clinical practice guidelines: care of the patient with accommodative and vergence dysfunctions. St Louis: American Optometric Association; 2010. pp. 4-23. https://www.aoa.org/documents/optometrists/CPG-18.pdf
2. Wick BC. Horizontal deviations. In: Amos JF, ed. Diagnosis and management in vision care. Boston: Butterworth-Heinemann; 1987. pp. 461-510.
3. García-Muñoz Á, Carbonell-Bonete S, Cacho-Martínez P. Symptomatology associated with accommodative and binocular vision anomalies. J Optom. 2014; 7: 178-192. doi: 10.1016/j.optom.2014.06.005
4. Bade A, Boas M, Gallaway M, Mitchell GL, Scheiman M, Kulp MT, et al. Relationship between clinical signs and symptoms of convergence insufficiency. Optom Vis Sci. 2013; 90: 988-995. doi: 10.1097/OPX.0000000000000012
5. Cooper J, Jamal N. Convergence insufficiency-a major review. Optometry. 2012; 83: 137-158. PMID: 23231437
6. Dwyer P. The prevalence of vergence accommodation disorders in a school-age population. Clin Exp Optom. 1992; 75: 10-18. doi: 10.1111/j.1444-0938.1992.tb01010.x

7. Paniccia SM, Ayala AR. Prevalence of accommodative and non-strabismic binocular anomalies in a Puerto Rican pediatric population. Optom Vis Perform. 2015; 3: 158-164. https://www.ovpjournal.org/uploads/2/3/8/9/23898265/ovp3-3_article_paniccia_web.pdf

8. Cacho-Martínez P, García-Muñoz Á, Ruiz-Cantero MT. Do we really know the prevalence of accomodative and nonstrabismic binocular dysfunctions? J Optom. 2010; 3: 185-197. doi: 10.1016/S1888-4296(10)70028-5

9. Menjivar AM, Kulp MT, Mitchell GL, Toole AJ, Reuter K. Screening for convergence insufficiency in school-age children. Clin Exp Optom 2018; 101: 578-584. doi: 10.1111/cxo.12661

10. Rouse M, Borsting E, Mitchell GL, Cotter SA, Kulp M, Scheiman M, et al. Validity of the convergence insufficiency symptom survey: a confirmatory study. Optom Vis Sci. 2009; 86: 357-363. doi: 10.1097/OPX.0b013e3181989252

11. Cacho-Martínez P, García-Muñoz A, Ruiz-Cantero MT. Diagnostic validity of clinical signs associated with a large exophoria at near. J Ophthalmol. 2013; 2013: 549435. doi: 10.1155/2013/549435

12. Kim YH. Binocular accommodative facility and vergence facility in university students with symptomatic convergence insufficiency. J Korean Ophthalmic Opt Soc. 2018; 23: 117-123. doi: 10.14479/jkoos.2018.23.2.117

13. Darko-Takyi C, Khan NE, Nirghin U. A review of the classification of nonstrabismic binocular vision anomalies. Optometry Reports. 2016; 5: 5626. doi: 10.4081/optometry.2016.5626

14. Cacho Martínez P, García Muñoz A, Ruiz-Cantero MT. Treatment of accommodative and nonstrabismic binocular dysfunctions: a systematic review. Optometry. 2009; 80: 702-716. doi: 10.1016/j.optm.2009.06.011

15. Scheiman M, Gallaway M, Frantz KA, Peters RJ, Hatch S, Cuff M, et al. Nearpoint of convergence: test procedure, target selection, and normative data. Optom Vis Sci. 2003; 80: 214-225. doi: 10.1097/00006324-200303000-00011

16. Maples WC, Savoy RS, Harville BJ, Golden LR, Hoenes R. Comparison of distance and near heterophoria by two clinical methods. Optom Vis Dev. 2009; 40: 100-106. https://cdn.ymaws.com/www.covd.org/resource/resmgr/ovd40-2/article_comparisonofdistance.pdf

~~17. Abraham LM, Kuriakose T, Sivanandam V, Venkatesan N, Thomas R, Muliyil J. Amplitude of accommodation and its relation to refractive errors. Indian J Ophthalmol. 2005; 53: 105-108. doi: 10.4103/0301-4738.16173~~

~~18. Jang JU, Park IJ, Jang JY. The distribution of near point of convergence, near horizontal heterophoria, and near vergence among myopic children in South Korea. Taiwan J Ophthalmol. 2016; 6: 187-192. doi: 10.1016/j.tjo.2016.07.001~~

17. Scheiman M. Wick B. Clinical management of binocular vision: heterophoric, accommodative, and eye movement disorders, 4th ed. Philadelphia: Lippincott Williams & Wilkins; 2014. pp. 36-76.

~~19~~18. Tsai LH, Lin DPC, Su JKC, Chang YS, Chen ST. The effects of myopia and AC/A measuring methodology on AC/A ratio outcome. Chung Shan Medical Journal. 2012; 23: 87-94. doi: 10.30096/CSMJ.201212.0003

~~20~~19. García A, Cacho P, Lara F. Evaluating relative accommodations in general binocular dysfunctions. Optom Vis Sci. 2002; 79: 779-787. doi: 10.1097/00006324-200212000-00010

~~21~~20. Weissberg EM. Diagnostic techniques. In: Weissberg EW, ed. Essential of clinical binocular vision. St.Louis: Butterworth-Heinemann; 2004. pp. 158-164.

~~22~~21. Gall R, Wick B, Bedell H. Vergence facility: establishing clinical utility. Optom Vis Sci. 1998; 75: 731-742. doi: 10.1097/00006324-199810000-00018

~~23~~22. Worrell BE Jr, Hirsch MJ, Morgan MW. An evaluation of prism prescribed by Sheard's criterion. Am J Optom Arch Am Acad Optom. 1971; 48: 373-376. PMID: 4931926

~~24~~23. Percival A. The prescribing of spectacles, 3rd ed. Bristol: John Wright & Sons; 1928. pp. 102-109. https://archive.org/details/b21287405/page/102

24. Benjamin WJ. Borish’s clinical refraction, 2nd ed. St. Louis: Butterworth-Heinemann; 2006. pp. 968-972.

~~25. Scheiman M. Wick B. Clinical management of binocular vision: heterophoric, accommodative, and eye movement disorders, 4th ed. Philadelphia: Lippincott Williams & Wilkins; 2014. pp. 52~~36~~-76.~~

~~26~~25. Cooper J, Duckman R. Convergence insufficiency: incidence, diagnosis, and treatment. J Am Optom Assoc. 1978; 49: 673-680. PMID: 355298

~~27~~26. Porcar E, Martinez-Palomera A. Prevalence of general binocular dysfunctions in a population of university students. Optom Vis Sci. 1997; 74: 111-113. doi: 10.1097/00006324-199702000-00023

~~28~~27. Wajuihian SO, Hansraj R. A review of non-strabismic accommodative-vergence anomalies in school-age children. Part 1: Vergence anomalies. Afr Vision Eye Health. 2015; 74: 1-10. doi: 10.4102/aveh.v74i1.32

~~29~~28. Gupta R, Sharma B, Anand R, Bawaria S, Dewada R. Association of asthenopia and convergence insufficiency in children with refractive error - a hospital based study. Int J Med Res Rev. 2013; 1: 222-227. http://medresearch.in/index.php/IJMRR/article/view/51/178

~~30~~29. Wajuihian SO. Is there an association between convergence insufficiency and refractive errors?. Afr Vision Eye Health. 2017; 76: 1-7. doi: https://doi.org/10.4102/aveh.v76i1.363

~~31~~30. Davis AL, Harvey EM, Twelker JD, Miller JM, Leonard-Green T, Campus I. Convergence insufficiency, accommodative insufficiency, visual symptoms, and astigmatism in Tohono O'odham students. J Ophthalmol. 2016; 2016: 1-7. doi: 10.1155/2016/6963976

~~32~~31. Schor CM. A dynamic model of cross-coupling between accommodation and convergence: simulations of step and frequency responses. Optom Vis Sci. 1992; 69: 258-269. doi: 10.1097/00006324-199204000-00002

~~33~~32. Convergence Insufficiency Treatment Trial Study Group. Randomized clinical trial of treatments for symptomatic convergence insufficiency in children. Arch Ophthalmol. 2008; 126: 1336-1349. doi: 10.1001/archopht.126.10.1336

~~34~~33. Sheedy JE, Saladin JJ. Association of symptoms with measures of oculomotor deficiencies. Am J Optom Physiol Opt. 1978; 55: 670-676. PMID: 747192

~~35~~34. O'Leary CI, Evans BJ. Double-masked randomised placebo-controlled trial of the effect of prismatic corrections on rate of reading and the relationship with symptoms. Ophthalmic Physiol Opt. 2006; 26: 555-565. doi: 10.1111/j.1475-1313.2006.00400.x

~~36~~35. Yu DS, Cho HG, Moon BY. Influence of different diagnostic criteria on frequency of convergence insufficiency. J Korean Ophthalmic Opt Soc. 2016; 21: 235-242. doi: 10.14479/jkoos.2016.21.3.235

~~37~~36. Gallaway M, Scheiman M, Malhotra K. The effectiveness of pencil pushups treatment for convergence insufficiency: a pilot study. Optom Vis Sci. 2002; 79: 265-267. doi: 10.1097/00006324-200204000-00013

~~38~~37. Sheedy JE, Saladin JJ. Phoria, vergence, and fixation disparity in oculomotor problems. Am J Optom Physiol Opt. 1977; 54: 474-478. PMID: 931014

~~39~~38. Horwood AM, Toor S, Riddell PM. Screening for convergence insufficiency using the CISS is not indicated in young adults. Br J Ophthalmol. 2014; 98: 679-683. doi: 10.1136/bjophthalmol-2013-304533

~~40~~39. Borsting E, Rouse MW, Deland PN, Hovett S, Kimura D, Park M, et al. Association of symptoms and convergence and accommodative insufficiency in school-age children. Optometry. 2003; 74: 25-34. PMID: 12539890

Journal Requirements:

(Requirements and Reviewer's comments are shown in *Italic*, line numbers pointed are number of revised manuscript)

When submitting your revision, we need you to address these additional requirements.

1. Please ensure that your manuscript meets PLOS ONE's style requirements, including those for file naming. The PLOS ONE style templates can be found at http://www.journals.plos.org/plosone/s/file?id=wjVg/PLOSOne_formatting_sample_main_body.pdf and http://www.journals.plos.org/plosone/s/file?id=ba62/PLOSOne_formatting_sample_title_authors_affiliations.pdf

Response to Journal requirements (RJ1): We have verified and revised the manuscript to meet the 'Manuscript body formatting guidelines' and 'Title, author, affiliations formatting guidelines'.

2. We noticed you have some minor occurrence of overlapping text with the following previous publication, which needs to be addressed: INSUFFI, CONVERGENCE. "Randomized clinical trial of treatments for symptomatic convergence insufficiency in children." Arch Ophthalmol 126.10 (2008): 1336-1349. In your revision ensure you cite all your sources (including your own works), and quote or rephrase any duplicated text outside the methods section. Further consideration is dependent on these concerns being addressed.

RJ2: The result of a check using the plagiarism detection software of 'www.copykiller.com' for 177 sentences of the submitted manuscript revealed a duplication rate of 1% compared with 'Arch Ophthalmol 126.10 (2008): 1336-1349' (Figure 1R below shows a part of the sample). Compared with other journals, the duplicated rate was 4% (Figure 2R below also shows a part of the sample). We have rechecked and rephrased the detected text, and have summarized these revisions in Table 1R:

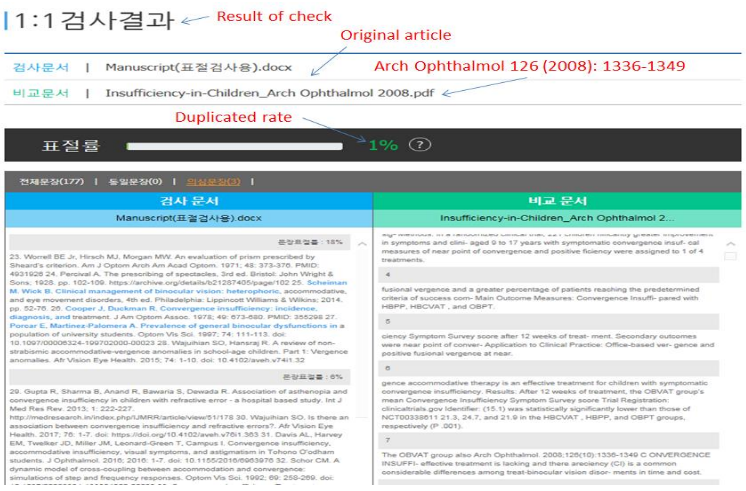

Figure 1R. Result of plagiarism detection for the submitted manuscript vs. Arch Ophthalmol 126 (2008): 1336-1349

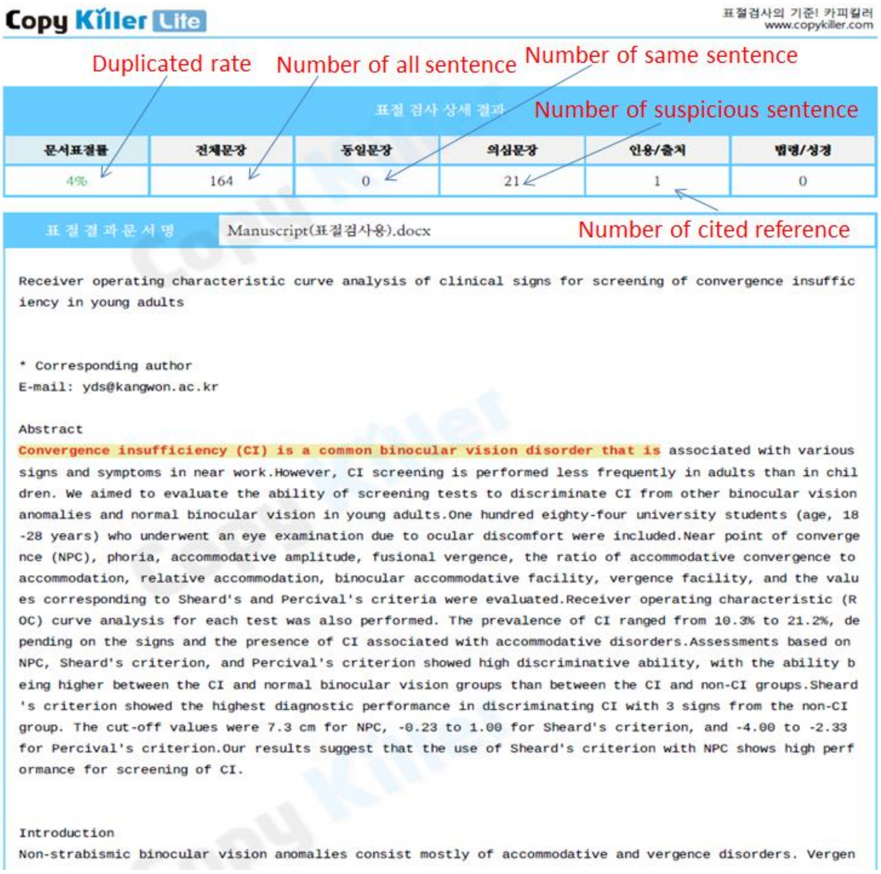
Copy Killer Lite

표절검사의 기준! 카피킬러
www.copykiller.com

Duplicated rate
Number of all sentence
Number of same sentence
Number of suspicious sentence
Number of cited reference

표절 검사 상세 결과

문서표절률	전체문장	동일문장	의심문장	인용/출처	법령/성경
4%	164	0	21	1	0

표절결과문서명: Manuscript(표절검사용).docx

Receiver operating characteristic curve analysis of clinical signs for screening of convergence insufficiency in young adults

* Corresponding author
E-mail: yds@kangwon.ac.kr

Abstract
Convergence insufficiency (CI) is a common binocular vision disorder that is associated with various signs and symptoms in near work.However, CI screening is performed less frequently in adults than in children. We aimed to evaluate the ability of screening tests to discriminate CI from other binocular vision anomalies and normal binocular vision in young adults.One hundred eighty-four university students (age, 18-28 years) who underwent an eye examination due to ocular discomfort were included.Near point of convergence (NPC), phoria, accommodative amplitude, fusional vergence, the ratio of accommodative convergence to accommodation, relative accommodation, binocular accommodative facility, vergence facility, and the values corresponding to Sheard's and Percival's criteria were evaluated.Receiver operating characteristic (ROC) curve analysis for each test was also performed. The prevalence of CI ranged from 10.3% to 21.2%, depending on the signs and the presence of CI associated with accommodative disorders.Assessments based on NPC, Sheard's criterion, and Percival's criterion showed high discriminative ability, with the ability being higher between the CI and normal binocular vision groups than between the CI and non-CI groups.Sheard's criterion showed the highest diagnostic performance in discriminating CI with 3 signs from the non-CI group. The cut-off values were 7.3 cm for NPC, -0.23 to 1.00 for Sheard's criterion, and -4.00 to -2.33 for Percival's criterion.Our results suggest that the use of Sheard's criterion with NPC shows high performance for screening of CI.

Introduction
Non-strabismic binocular vision anomalies consist mostly of accommodative and vergence disorders. Vergen

Figure 2R. Result of plagiarism detection for the submitted manuscript vs. other articles

Table 1R. Summary of plagiarism detection and the revisions made to the manuscript.

Detected phrases	Revision (quotation and rephrase)
Line 15 & Line 43–44[†]: Convergence insufficiency (CI) is a common binocular vision disorder that is	Convergence insufficiency (CI) is a common dysfunction of binocular vision that is
Line 46: signs of CI include exophoria that is greater at near than at distance	We have quoted '[1]' at end of related sentence (line 48).
Line 49: not all patients with CI have all of these	This part had quoted '[1, 4]' at end of sentence.
Line 57–58: ROC curve analysis was used to evaluate the ability of	ROC curve analysis was used to assess the performance of

Line 96–99: This study was approved by the Kangwon national university institutional review board and adhered to the tenets of the Declaration of Helsinki.	This study was approved by the Kangwon National University Institutional Review Board (KWNUIRB-2019-02-001-002) with waiver of the informed consent for the retrospective collection of clinical data and adhered to the tenets of the Declaration of Helsinki. Please note this text is in the methods section.
Line 187–188; line 223–227	These are notes not related to plagiarism.
Line 192–193; line 194–195; line 199–200	We have not considered revising the text because these parts were related to the methods section.
Line 210–211: are shown in Table 2. Subjects were divided into three groups	Expressions not related to plagiarism.
Line 256: the area under the curve (AUC) in ROC curve analysis	Expressions not related to plagiarism.
Line 258: in the notes for Tables 1, 2, and 3.	Expressions not related to plagiarism.
Line 267–268: the sensitivity, specificity, positive likelihood ratio (LR+), and negative likelihood ratio (LR-) for each	We have not considered this general phrasing as plagiarism.
Line 347–348: subjects with large near exophoria and moderate to severe symptoms	This part has the citation '[11]' at end of the sentence.
Line 408: one limitation of this study is the lack of	We have not considered this general phrasing as plagiarism.
Line 410–411: no further association between the severity of the clinical signs and	This part has the citation '[4]' at end of the sentence.
Line 411–412: On the other hand, other studies have shown an association between	This part has the citations '[9, 40]' at end of the sentence.

† Lines on submitted PONE-D-19-17984.

3. Please provide additional details regarding participant consent. In the ethics statement in the Methods and online submission information, please ensure that you have specified (1) whether consent was informed and (2) what type you obtained (for instance, written or verbal, and if verbal, how it was documented and witnessed). If the need for consent was waived by the ethics committee, please include this information.

RJ2: 'with waiver of the informed consent for the retrospective collection of clinical data' was added to line 103.

Comments to the author:

1. Is the manuscript technically sound, and do the data support the conclusions?

Reviewer #1: Partly
Reviewer #2: Partly
Reviewer #3: Yes

Authors' Response: We have revised the manuscript according to the reviewers' comments.

---

2. Has the statistical analysis been performed appropriately and rigorously?

Reviewer #1: Yes
Reviewer #2: No
Reviewer #3: N/A

Authors' Response: We have made revisions as mentioned for response 29 to reviewer #2 (R29R#2), R33R#2, R35R#2, and R4R#3.

---

3. Have the authors made all data underlying the findings in their manuscript fully available?

Reviewer #1: Yes
Reviewer #2: Yes
Reviewer #3: Yes

Authors' Response: Thank you for your positive comments.

---

4. Is the manuscript presented in an intelligible fashion and written in standard English?

Reviewer #1: Yes
Reviewer #2: No
Reviewer #3: No

Authors' Response: We have revised all the specific errors pointed out by the reviewers. The revised manuscript has also been edited by a native English speaker.

---

5. Review Comments to the Author

Reviewer #1:

• *SUMMARY:*
The article provides an analysis of differentiating which clinical tests that are best to be performed to diagnose a common binocular vision anomaly, i.e. the convergence insufficiency (CI). The complexity of the anomaly makes practitioners confused and sometimes missed out in detecting and diagnosing CI. As such, this article highlights the important clinical tests and criteria that could help practitioner by performing the Receiver operating characteristic (ROC) curve analysis. While the ROC analysis were well performed, there are a few issues that needs to be addressed as stated below.
Response 1 to Reviewer #1 (R1R#1): Thank you for these positive comments. We have carefully revised the initial manuscript based on your constructive and helpful comments.

• *FEEDBACK:*

1. Materials & Methods Section
• *Line 99*
• *Please also clarify if participants recruited have not had any vision therapy or eye exercise treatment prior to the enrollment as research participant.*
R2R#1: ‘Participants had not previously undergone any vison therapy or eye exercise treatment.’ was added in lines 104-105 of the revised manuscript.

• *Line 112*
• *The authors should detailed the NPC measurement whether it was done in a free space using accommodative fixation target or using the RAF ruler.*
• *MAJOR: Target used for the test (20/50) was too large for the 20/20 research participants group. Using this target will not exert the best binocular vergence ability of the participants and would affect the whole test outcomes. Please clarify.*
R3R#1: Thank you for this detailed comment. 1) ‘In free space’ and ‘on a Gulden fixation stick’ were added to the methods section. 2) A large target may not be able to achieve the best binocular vergence ability, as the reviewer has pointed out. However, various targets can be used to measure NPC, such as near accommodative targets (different sizes in the range of 20/25 to 20/200), pencil tip, penlight, and penlight with red/green glasses [R1]. Several studies have stated the differences in the NPC with different target types are not likely to be clinically significant [R2], and the detail of the accommodative target is also irrelevant as there are no significant differences between a fingertip, pencil tip, or N5 letter presented in free space [R3]. In addition, it has been suggested that a target with fine detail should be avoided, otherwise patients often confuse blur with diplopia [R4]. Therefore, we chose to use the isolated 20/50 target to measure NPC [R5, R6].
Reference:
[R1] Carlson NB, Kurtz D. Clinical procedures for ocular examination, 3rd ed. New York: The McGraw-Hill; 2004. pp. 50–51.
[R2] Siderov J, Chiu SC, Waugh SJ. Differences in the nearpoint of convergence with target type. Ophthalmic Physiol Opt. 2001; 21(5):356-360.

[R3] Adler PM, Cregg M, Viollier AJ, Margaret Woodhouse J. Influence of target type and RAF rule on the measurement of near point of convergence. Ophthalmic Physiol Opt. 2007; 27(1):22-30.
[R4] Elliott D. Clinical procedures in primary eye care, 3rd ed. Edinburgh: Butterworth-Heinemann; 2007. pp. 188–190.
[R5] Scheiman M. Wick B. Clinical management of binocular vision: heterophoric, accommodative, and eye movement disorders, 4th ed. Philadelphia: Lippincott Williams & Wilkins; 2014. pp. 43–44.
[R6] Ostadimoghaddam H, Hashemi H, Nabovati P, Yekta A, Khabazkhoob M. The distribution of near point of convergence and its association with age, gender and refractive error: a population-based study. Clin Exp Optom. 2017; 100(3):255-259.

• *The authors should specify if the procedures were performed by a sole researcher or a group of researchers. Please also state if the procedures were done in the same order for each participant as some binocular vision assessments are invasive, i.e. will likely introduce fatigue to participants if it is not controlled. This too would affect the whole test outcomes and data collected.*
R4R#1: Thank you for pointing out these details that we had missed. 'All measurements were taken in a general clinical room by the same examiner, who performed all tests within approximately 30 minutes, using the same methodology.' were added to lines 106-108.

2. Results Section
- *Line 207*
- *Instead of writing the refractive errors and classify them for 'each eye', I would suggest that the authors change the way of writing this as per participant, as convergence insufficiency is a binocular anomaly and not a monocular phenomenon.*

R5R#1: We had only presented the characteristics of the participants' refractive errors in this part. We agree with the reviewer that it would be good to present refractive errors based on both eyes per participant. Therefore, we have added 'Myopia was present in 125 participants (67.9%), hyperopia in six participants (3.3%), emmetropia in 42 participants (22.8%), mixed anisometropia in four participants (2.2%), simple myopic anisometropia in four participants (2.2%), and simple hyperopic anisometropia in three participants (1.6%).' to lines 237-240.

- *Line 219*
- *It would be beneficial if the authors add in the normative data (e.g.: Morgan's Normative Data on Binocular Vision) in Table 2 for the readers to understand the distribution of the data against the normative data used clinically.*

R6R#1: Normative values have been included in the method of each test. References '[17, 24]' related to Morgan's normative data have been included in lines 127, 138, 148, 159, 168, 175, 182, and 188.

- *Line 269*
- *The NPC cut-off written in the paragraph was >7.3cm, different than what is stated in Table 5 (>7.2cm). Please double-check.*

R7R#1: We have rewritten 7.3 cm to 7.2 cm.

• The sensitivity and specificity for ROC analysis are usually expressed in percentage with 95% confidence interval. The authors may want to revise how they written this to make it more readable for other researchers.
R8R#1: We think that this choice depends on the authors' preference or statistics program. Sensitivity is shown on the *y* axis from 0 to 1 (0–100%), and 1-specificity is shown on the *x* axis from 0 to 1 (0–100%). The IBM SPSS statistics program used in this study provides a scale of 0–1. The area under curve (AUC) also has a scale of 0–1. Scales of 0–1 are seen frequently in PLoS ONE [R1].
Reference:
[R1] deCastro BR. Cumulative ROC curves for discriminating three or more ordinal outcomes with cutpoints on a shared continuous measurement scale. PLoS ONE 2019; 14(8): e0221433.

3. Discussion Section
• Although the participants' groupings and parameters are complicated but the authors managed to write and explain in a good flow. I hope to see a more analytical explanation when discussing the outputs and its relationship to what have been the current knowledge in diagnosing convergence insufficiency. Some comments I have stated above may or may not change the outputs and interpretation of the results.
R9R#1: Thank you for these detailed and important comments that help us to improve the submitted manuscript. We recognize that the technical expressions are sometimes limited. However, we have done our best to provide a sophisticated explanation and the unclear parts have been revised.

4. Grammatical errors (see attachment)
R10R#1: All have been revised according to the reviewer' comments as follows:
- Line 95 & 97: "," has replaced by "=" in line 98.
- Line 107 & 108: 'national university institutional review board' has replaced by 'National University Institutional Review Board'; 'corporation' has replaced by 'Corporation' in lines 102, and 114.
- Line 157: '±2.0 D' has replaced by '±2.00 D' in 177.
- Line 311: 'vison' has replaced by 'vision' in line 345.

========================

Reviewer #2:

Generally, the Introduction is well-written. Within the Methods there are missing details which mean that the study could not be reproduced. The Results is the weakest section. It is not clear which participants are included in which groups and the names of the groups were changed throughout the results. Please see the comments below for details. It was difficult to follow the discussion due to the concerns regarding the results. I would recommend making those changes first to allow the reviewer to make more informed comments regarding the discussion.
R1R#2: Thank you for your positive comments regarding the Introduction. The weakest sections (Methods and Results) have been revised according to the reviewer's detailed comments, and we have also rechecked the discussion. These comments are very constructive and valuable, and

they have helped us to improve the clarity of the manuscript, specifically in terms of our methodology and the goal of this manuscript.

Introduction
44 – delete 'common'. There is no evidence provided and later on the prevalence of CI is covered with references.
R2R#2: We have deleted 'common' from the introduction and from the abstract.

46-8 – no references for the signs of CI
R3R#2: The signs of CI are well known. However, we have included a reference [1] for the readers to understand the signs for CI.

65-67 – study 11 not clearly presented. Did their participants have CI? Were they symptomatic? Was ROC curve analysis used? It is not clear how this study is relevant.
R4R#2: Considering that CI is a nonstrabismic binocular anomaly associated with a large near exophoria, study [11] aimed to analyze the diagnostic validity of accommodative and binocular tests in a sample of patients with a large near exophoria with moderate to severe symptoms, and to analyze their diagnostic validity by means of ROC analysis, sensitivity, specificity, and likelihood ratios. Therefore, 'A previous study used ROC analysis to investigate the diagnostic validity of the clinical signs of CI in participants aged 19 to 35 years with either symptomatic large exophoria and normal heterophoria, or low visual discomfort at near [11]; it reported that NPC and BAF tests showed the best diagnostic accuracy for discriminating between the two groups' has been included in lines 65-69.

69 – did study 12 test NPC? This was the test that showed best diagnostic ability in the other studies. I would assume this would be tested in this study investigating CI.
R5R#2: Study [12] focused on only BAF and VF. In this study, participants were classified into the CI and normal groups using the Convergence Insufficiency Symptom Survey (CISS). These descriptions have been included in lines 67-69.

77-84 This is an unclear p/g. The first approach requires the analysis of signs measured by each test, as does the second approach. It is not clear what the difference is. Do you mean the final 'approach' rather than 'stage'? The 3rd approach also needs to be explained more clearly.
R6R#2: Thank you for these detailed comments. The first approach is used to distinguish CI from accommodative dysfunction. We have replaced 'stage' with 'approach'. As the 3rd approach relates to the lines 80-84, we linked the first and last sentences.

86 – *it is not clear why fusional vergence has been explicitly stated, whilst the others are all put under 'signs'.*
R7R#2: In vergence disorders such as CI, the measurement of fusional vergence is an important in evaluating ability to maintain single binocular vision. Therefore, in CI screening, fusional vergence should be evaluated due to one of signs related to CI. We have added 'related to ability to maintain single binocular vision' to line 90.

Methods

In the introduction it was stated that the students had 'visited for primary eye care'. That should appear in the method section instead and it should be stated whether this was in a hospital setting. It is not clear how they were recruited. Was the project advertised to seek students with ocular discomfort? Or were University students identified within the primary eye care and asked to partake?

How was it determined that these students had ocular discomfort? Were they asked any questions regarding this?

R8R#2: Thank you for pointing out these details that we missed. 'Participating students voluntarily visited a university eye clinic center for primary eye care due to blurred vision, eyestrain, and visual discomfort.' was added to lines 96-101. History taking related to ocular symptoms has been performed to find out the purpose of student's visit as general process for eye examination.

96 - Were set questions asked about the ocular discomfort?
R9R#2: The Suggested Questions for Patient History described in reference [1] were followed. The reference was cited in line 107.

105 – replace 'by an ocular motility and' with 'by ocular motility testing and'
R10R#2: We have revised the text according to the reviewer's comment.

107 - It is not clear how the phoropter was used for binocular vision tests
R11R#2: The phoropter is an instrument invented in the early 1900s and used to measure the ametropias, phoria, and the amplitude of accommodation of the eyes. The power of lenses in the phoropter can be modified in steps of 0.25 D. It is commonly used by eye care professionals during an eye examination. We think that a description of how to use it is not necessary.

108 – tests were performed at distance 'or' near – should this be 'and'?
R12R#2: We have revised the text according to the reviewer's comment.

112-6 – was the distance for NPC recorded in cm? How was the speed of the target controlled to 5cm/s? 'at the eye level and between the both eyes' should be 'between both eyes and at eye level'.
R13R#2: '(cm)' in line 124, 'approximately' in line 126, and 'between both eyes and at eye level' in line 127 were included or revised. The speed of the target was approximated considering the rate of the patient's reaction.

122 – how was the speed controlled to 2PD per sec?
When measuring the accommodative amplitude, how was the speed controlled? What were the instructions? Were they told to report when the target first became blurred or when it was so blurred they could read the letters? The last word 'accommodation' should state '(in meters).
R14R#2: Speeds that were either too slow or too fast could affect the measured values, so we tried to regulate the speed for measurement to rule out this affect. 'the subject reported when the

target first became blurred while moving’ in lines 145-149, ‘(cm)’ ‘approximately’ in line 146, and ‘(m)’ in line 148 were included in the revised text.

139 – this reference no. 18 seems inappropriate. Is it to justify the method being used? The use of references need to be considered as no. 17 (129) also appeared inappropriate.
R15R#2: References [17] and [18] were replaced with [25] (renumbered as [17]). The page of reference [25] was changed from pp. 52-76 to pp. 36-76.

142 – the word ‘prism’ is missing
R16R#2: We have revised the text accordingly.

143 – Relating back to my point re. 139, it would be more appropriate to have a reference here to state why testing was done at near first.
R17R#2: Reference [17] was cited in line 153.

The word AC/A should always be followed by ‘ratio’. Why was 1D used? It is typically measured using 3D. Either explain or use a reference to justify the method. The distance was divided by 2.5D, presumably because the test was conducted at 40cm, but this should be stated somewhere. There was also not enough detail on how the lenses were introduced and how IPD was measured? Was this all done using the phoropter?
R18R#2: Thank you for your detailed comments. AC/A means the ratio of AC to A. Therefore, we think that ‘ratio’ is not necessary. We have defined ‘Gradient AC/A’ as ‘Gradient AC/A ratio (AC/A)’ in line 164. Gradient AC/A can be determined by adding +3D to -3D. We used +1.00D considering the addition of plus power (less minus power) in a binocular vision prescription. In a phoria prescription, the preference of the prescription is in order of vision training (VT), addition of plus power (as the same maximum plus power for maximum visual acuity), prism prescription, and so on. IPD was measured with a PD meter. Reference [17] was included. Lines 166–167 were partly revised.

153 – Was the phoropter used to change lens strengths? Normative values should be presented here, and for all the other tests used.
R19R#2: In the ‘General procedure’ section, the use of the phoropter has been stated. As mentioned in the response of R11R#2, the lens power in the phoropter can be changed by steps of 0.25 D. We have provided normative values, as requested.

Was BAF tested at 40cm? Normative values from the literature would also be helpful.
R20R#2: BAF was tested at 40cm. 40cm was included in line 177. Normative values were presented from thein literature [17, 24].

Vergence facility testing is the main concern of the method section. The method described does not test VF. This would require a BI and a BO prism, yet the current study used a BI and BU prism. Is this a typing error? The subject should also be reporting when the target becomes single, not when they note clear vision.
R21R#2: Thank you for these valuable comments. This is a typing error. ‘clear vision’ has been replaced with ‘single vision’.

The method used to calculate Percival's criterion is not clearly presented.
R22R#2: Percival's criterion was stated in line 194-197. In these lines, prism = (1/3) (greater of the two fusional vergence) – (2/3) (lesser of the two fusional vergence) was described.

179-83 – What is Morgan's? Is this a reference? It is not in the reference list either. So were all participants used and categorised into one of the 10 diagnoses? It is not clear what the expected criteria is for each test? Do you mean the expected normative values? These could have been presented when describing each of the tests. It is not clear what modification were made from the study by Scheiman and Wick.
R23R#2: Morgan's refers to Morgan's table of expected findings for each test. Morgan's expected values for each test were included in references [17, 24]. Normative values of each test have been included in the methods section. This 'which were modified from' is a describing error. 'which were modified from' have been replaced with 'which referred to'.

Table 1 – '1 and 2 or 3'…does this mean you need both signs 1 and 2, or just sign 3? Or does this mean you need sign 1 as well as sign 2 or 3? If it is the first option, then perhaps 'signs 1 and 2, or sign 3' would be better? If it is the second option, perhaps put this as 'presence of sign 1 and sign 2 or 3'.
R24R#2: Table 1 has revised as 'sign 1 and sign 2 or 3'.

Table 1 – What is meant by a 'normal phoria'? What counts as 'reduced' PFV and NFV? Can abbreviate binocular accommodative facility under AI. Should BAF be more than 13 cpm in AE? Is a reference required for this table if it has been taken and modified from another source?
R25R#2: We have included additional explanations to improve the clarity of the table. References have been cited as stated in R23R#2. BAF should be less than 13 cpm in AE due to fail for +2.00D.

194-204 – Which 3 groups were compared? It has not been stated that the subjects were put into 3 groups. I had assumed subjects were put into the 10 diagnostic categories based on the information that has been provided. Should the sentence on specificity and sensitivity should come before it was stated that the ROC was calculated because the sensitivity values are used for the ROC? It would be useful to state this and 1-specificity are used to plot the curve. Perhaps I am mistaken but I think the 'cut-off' is the value that you use to decide what test result indicates whether or not someone has CI, and therefore would affect the sensitivity and specificity, and therefore would affect the curve. How was the AUC calculated? It would be useful to indicate that a AUC value of 1 and 0.5 are important and why.
R26R#2: The three groups were all CI, binocular vision anomaly except CI (BVA), and normal binocular vision (NBV). The groups are described in lines 202-203. 'by plotting sensitivity on the y axis as a function of 1 – specificity on the x axis' have added to line 220. 'The cut-off was also identified for each test with the largest AUC which was significantly greater than 0.50.' has been added to lines 228-229.

Results

Were all the subjects with a refractive error wearing their refractive correction for testing?
R27R#2: ‘after wearing refractive correction’ has been added to lines 241-242 to clarify this.

211 – Here it is stated what the 3 groups are but this should have appeared in the method section. The groups are unclearly presented. What is meant by ‘all CI’? Is this anyone that fell into the CI category in Table 1? Are those in ‘BVA’ anyone that falls into any other diagnosis based on Table 1? I assume NBV are those that have nothing wrong and it might be less confusing to refer to them as the control group. The abbreviation BVA is also confusing as that typically stands for binocular visual acuity
R28R#2: In relation to the answer given in R26R#2, a description of the three groups has been noted to lines 203-208 of the method section as follows: ‘Subjects groups were classified into three groups according to Table 1: All CI including CI with 2 signs and CI with an accommodative dysfunction, other binocular vision anomaly (BVA) except CI, and normal binocular vision (NBV). All CI were also classified into CI with 3 signs (CI3), CI with 2 signs and an accommodative dysfunction (CI3AD), and CI with 2 signs (CI2).’ The abbreviation BVA was determined under our careful consideration.

Table 2 – The AA for All CI is reported to be 1.00D – is this correct? The significance results do not match up to those reported in the text on page 10. E.g. AA was reported to be significant on 214 but here it is not significant. NPC is reported to be significant in the table but is not reported on page 10. Phoria at distance in the table states that c is significant greater than b, but on page 10 it states that there was a significant difference between all CI and other BVA. These all need to be checked.
R29R#2: Thank you for this detailed check of the data. 10.90 D is the correct AA for all CI; we have revised the table 2 to reflect this. We have deleted AA and added NPC in line 245. There was a significant difference between all CI and other BVA, and other BVA and NBV in distance phoria and distance NFV. The text has been revised to make this clear.

229 – ‘non-strabismic binocular vision anomalies’ – does this refer to everyone in all CI and other BVA but excluding those in NBV? It is confusing as the terms keep interchanging. On line 232 is the NBV group now referred to as the ‘normal group’?
R30R#2: ‘non-strabismic binocular vision anomalies’ means all CI and other BVA except NBV. ‘non-strabismic’ was deleted to eliminate confusion in line 261 and from the title of Table 3. "normal group" has been replaced with "NBV" to avoid confusion in line 264.

232-38 Within this p/g indicate CI2AD etc so that it matches with the terms used in Table 3.
R31R#2: The paragraph has been revised to use NBV, CI2, CI + AI, CI + AE, CI3, and CI2AD so that it matches with the terms used in Table 3.

Table 3 – Again it is unclear who fits within the various groups. Does the ‘non-CI’ group contain everyone in the ‘other BVA’ group? Looking at the numbers it looks like this could be those in ‘other BVA’ and ‘NBV’. Does AD include anyone of those listed with AE and AI? I would assume so but the numbers don’t add up correctly. Why is FVD and accommodative infacility not included in this table?

R32R#2: 'Non-CI corresponding to CI is represented in the non-CI group.' has been included in Table 3 to improve its clarity. All (including NBV) except classified CI (CI3, CI3AD, CI2, CI2AD) have been classified into the non-CI group. In the case of this study, FVD and accommodative infacility was not present.

246-53 – 'for each CI test' is unclear. Firstly, it would be better to just refer to them as 'tests' rather than 'CI tests' and secondly, they are not all included in Table 4. Again it is stated 'for each test' yet Figure 1 only shows the ROC curves for NPC, Sheard and Percival. State why only particular tests are presented in Table 4 and figure 1. I assume 'for diagnostic tests excluded in Table 1' refers to Sheard and Percival? This should be explicitly stated, rather than requiring the reader to go back and work this out.

R33R#2: 'ROC curve analysis was performed for the tests shown in Table 2. The results of the AUC for NPC, Sheard's and Percival's criterion with $p < 0.05$ and AUC > 0.5 in 95% confidence interval ranges, and PFV, NFV, and AC/A including the diagnostic criteria in Table 1 are shown in Table 4' has been added to line 278-281. 'Fig 1 shows ROC curves for NPC, Sheard's and Percival's criterion' have been included in 281-282. The reason for 'why only particular tests are presented in Table 4 and figure 1' is that other tests except tests presented in Table 4 and Figure 1 were not significant and tests resented in Table 4 and Figure 1 were significant and high priority for CI screening (Figure R1).

<table>
<tr><th>For NPC, Sheard's, and Percival's criterion (Fig 1)</th><th>For all tests</th></tr>
<tr><td>
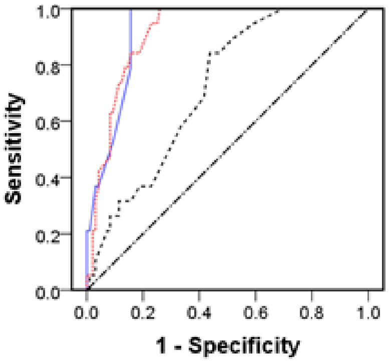

A. CI3 + NBV
</td><td>
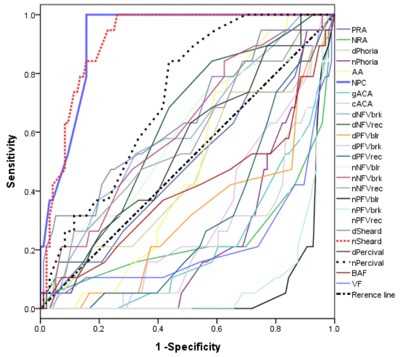

A. CI3 + NBV
</td></tr>
<tr><td>
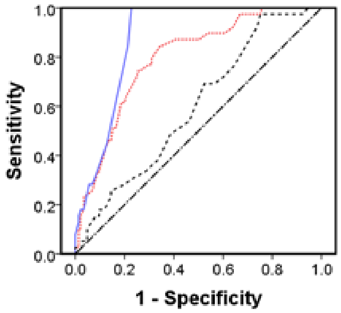

H. CI2AD + non-CI
</td><td>
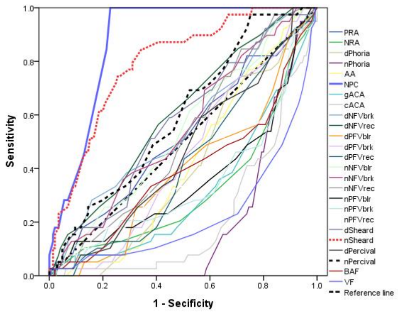

H. CI2AD + non-CI
</td></tr>
</table>

Figure R1. Example: comparison of ROC curves for particular tests (Fig 1) and all tests.

253 – ‘AUCs were greater for NBV than for non-CI’. In an earlier comment I had stated that it seemed that ‘non-CI’ included those in ‘other BVA’ and ‘NBV’, yet now it is suggested that this is not the case.
R34R#2: As in R30R#2 and R32R#2, when CI is classified as CI3, CI3AD, CI2, and CI2AD, respectively, the non-CI group includes all (other BVA and NBV) those except for the classified CI, respectively. For an example, Table 4 represents the ability to discriminate CI from CI3 + NBV, and discriminate CI from CI3 + non-CI.

Table 4 – How was it chosen which tests to present here? For NFV, how was it chosen whether to present break or recovery point and near or distance result? It is not clear what is meant by each of the ‘screening for various CI conditions’, e.g. are you comparing the groups, so CI3 versus NBV? Etc. The paragraph from 267 suggests you are combining the stated groups but it is not clear why you would be doing this.
R35R#2: We chose the tests to present here based on 1) Fig 1 and tests having $p < 0.05$ and AUC > 0.5 from all tests in Table 2, and 2) PFV, NFV, and AC/A including diagnostic criteria in Table 1. NFV for distance has been represented in a footnote in Table 4. ‘screening for various CI conditions’ has been revised to 'Ability to discriminate CI" to improve the clarity of Table 4. To further reduce confusion, the first column of Table 4 has also been revised.

Figure 1 – It is not clear which is the dotted and dashed line. Perhaps a coloured figure would be more appropriate.
R36R#2: Figures have been revised and are now represented by colors.

301 – should state ‘was also evaluated in NBV and non-CI conditions’?
R37R#2: We have corrected the text to ‘was also evaluated in NBV and non-CI conditions’.

308 – the accommodative conditions were abbreviated earlier. Also, what about accommodative infacility?
R38R#2: ‘accommodative insufficient (AI)’ has been changed to ‘AI’, ‘accommodative infacility’ had not been abbreviated.

311-2 – This sentence does not seem to link with the previous sentence.
R39R#2: This sentence has been corrected to ‘CI should also be distinguished from conditions such as normal vision, diverse vergence and accommodative disorders, and other binocular anomalies.’

327 – unclear why discriminating CI from abnormal rather than normal groups.
R40R#2: ‘from abnormal groups combined with other binocular anomalies’ has been added to lines 361-356 to clarify this.

========================

Reviewer #3:

This study was aimed to evaluate the ability of screening tests to discriminate convergence insufficiency, that is very important issue in the fields of heath science and related areas. The authors are presenting interesting results, however the manuscript should be improved:
R1R#3: Thank you for these positive comments. We have revised the manuscript according to your comments.

1. More information about criteria to select the participants should be added in the materials method section.
R2R#3: We have rechecked the manuscript, and have revised the unclear parts of the text. To summarize the inclusion criteria of participants, participants were university students, voluntarily visited with ocular discomfort, had no previous vision therapy or eye exercise treatment, no ocular disease and surgery, and did not have amblyopia and strabismus. These criteria have been added to line 95-101.

2. Have research group got the informed consents from study subjects, that information should be clearly indicated in the paper
R3R#3: We have clarified this by adding 'with waiver of the informed consent for the retrospective collection of clinical data' to lines 103-104.

3. Why did author consider the significantly difference when A p-value of ≤ 0.05, is that should be A p-value of < 0.05?
R4R#3: We have replaced 'A p-value of ≤ 0.05' with 'A p-value of < 0.05'.

4. The Table 1: is the criteria made by the authors, or from others? If from others these should be some citations
R5R#3: The criteria of Table 1 were obtained from Scheiman and Wick's study [17] and the expected criteria for each test [24]. This is explained in lines 181-183 in the submitted manuscript (208-210 in the revised manuscript).
Reference:
[17] Scheiman M. Wick B. Clinical management of binocular vision: heterophoric, accommodative, and eye movement disorders, 4th ed. Philadelphia: Lippincott Williams & Wilkins; 2014. pp. 36-76.
[24] Benjamin WJ. Borish's clinical refraction, 2nd ed. St. Louis: Butterworth-Heinemann; 2006. pp. 968-972.

5. Why the figure 1 does not have the legend?
R6R#3: The legend for Figure 1 can be found in lines 296-299. The legend has been placed in the text near to the first mention of Figure 1, as per the PLOS ONE guidelines.

6. English errors and writing style should be checked by native English speakers
R7R#3: Our manuscript has been checked by Editage for native English language editing.

- The end of response to reviewers' comments –
