# Supplementary material for: Receiver operating characteristic curve analysis of clinical signs for screening of convergence insufficiency in young adults
Source: PLoS One. 2020 Jan 24;15(1):e0228313. doi: 10.1371/journal.pone.0228313 (PMC6980576; doi:10.1371/journal.pone.0228313)
Supplement: Attachment — Submitted filename: Response to Reviewers.docx [file pone.0228313.s003.docx]

Journal Requirements:

(Requirements and Reviewer’s comments are shown in *Italic*, line numbers pointed are number of revised manuscript)

*When submitting your revision, we need you to address these additional requirements.*

*1. Please ensure that your manuscript meets PLOS ONE's style requirements, including those for file naming. The PLOS ONE style templates can be found at http://www.journals.plos.org/plosone/s/file?id=wjVg/PLOSOne_formatting_sample_main_body.pdf and http://www.journals.plos.org/plosone/s/file?id=ba62/PLOSOne_formatting_sample_title_authors_affiliations.pdf*

Response to Journal requirements (RJ1): We have verified and revised the manuscript to meet the ‘Manuscript body formatting guidelines’ and ‘Title, author, affiliations formatting guidelines’.

*2. We noticed you have some minor occurrence of overlapping text with the following previous publication, which needs to be addressed: INSUFFI, CONVERGENCE. "Randomized clinical trial of treatments for symptomatic convergence insufficiency in children." Arch Ophthalmol 126.10 (2008): 1336-1349. In your revision ensure you cite all your sources (including your own works), and quote or rephrase any duplicated text outside the methods section. Further consideration is dependent on these concerns being addressed.*

RJ2: The result of a check using the plagiarism detection software of ‘www.copykiller.com’ for 177 sentences of the submitted manuscript revealed a duplication rate of 1% compared with ‘Arch Ophthalmol 126.10 (2008): 1336-1349’ (Figure 1R below shows a part of the sample). Compared with other journals, the duplicated rate was 4% (Figure 2R below also shows a part of the sample). We have rechecked and rephrased the detected text, and have summarized these revisions in Table 1R:

Figure 1R. Result of plagiarism detection for the submitted manuscript vs. Arch Ophthalmol 126 (2008): 1336-1349

Figure 2R. Result of plagiarism detection for the submitted manuscript vs. other articles

Table 1R. Summary of plagiarism detection and the revisions made to the manuscript.

| Detected phrases | Revision (quotation and rephrase) |
| --- | --- |
| Line 15 & Line 43–44^†^: Convergence insufficiency (CI) is a common binocular vision disorder that is | Convergence insufficiency (CI) is a common dysfunction of binocular vision that is |
| Line 46: signs of CI include exophoria that is greater at near than at distance | We have quoted ‘[1]’ at end of related sentence (line 48). |
| Line 49: not all patients with CI have all of these | This part had quoted ‘[1, 4]’ at end of sentence. |
| Line 57–58: ROC curve analysis was used to evaluate the ability of | ROC curve analysis was used to assess the performance of |
| Line 96–99: This study was approved by the Kangwon national university institutional review board and adhered to the tenets of the Declaration of Helsinki. | This study was approved by the Kangwon National University Institutional Review Board (KWNUIRB-2019-02-001-002) with waiver of the informed consent for the retrospective collection of clinical data and adhered to the tenets of the Declaration of Helsinki.  Please note this text is in the methods section. |
| Line 187–188; line 223–227 | These are notes not related to plagiarism. |
| Line 192–193; line 194–195; line 199–200 | We have not considered revising the text because these parts were related to the methods section. |
| Line 210–211: are shown in Table 2. Subjects were divided into three groups | Expressions not related to plagiarism. |
| Line 256: the area under the curve (AUC) in ROC curve analysis | Expressions not related to plagiarism. |
| Line 258: in the notes for Tables 1, 2, and 3. | Expressions not related to plagiarism. |
| Line 267–268: the sensitivity, specificity, positive likelihood ratio (LR+), and negative likelihood ratio (LR-) for each | We have not considered this general phrasing as plagiarism. |
| Line 347–348: subjects with large near exophoria and moderate to severe symptoms | This part has the citation ‘[11]’ at end of the sentence. |
| Line 408: one limitation of this study is the lack of | We have not considered this general phrasing as plagiarism. |
| Line 410–411: no further association between the severity of the clinical signs and | This part has the citation ‘[4]’ at end of the sentence. |
| Line 411–412: On the other hand, other studies have shown an association between | This part has the citations ‘[9, 40]’ at end of the sentence. |

^†^ Lines on submitted PONE-D-19-17984.

*3. Please provide additional details regarding participant consent. In the ethics statement in the Methods and online submission information, please ensure that you have specified (1) whether consent was informed and (2) what type you obtained (for instance, written or verbal, and if verbal, how it was documented and witnessed). If the need for consent was waived by the ethics committee, please include this information.*

RJ2: ‘with waiver of the informed consent for the retrospective collection of clinical data’ was added to line 103.

Comments to the author:

*1. Is the manuscript technically sound, and do the data support the conclusions?

Reviewer #1: Partly
Reviewer #2: Partly
Reviewer #3: Yes*

Authors’ Response: We have revised the manuscript according to the reviewers’ comments.

*2. Has the statistical analysis been performed appropriately and rigorously?

Reviewer #1: Yes
Reviewer #2: No
Reviewer #3: N/A*

Authors’ Response: We have made revisions as mentioned for response 29 to reviewer #2 (R29R#2), R33R#2, R35R#2, and R4R#3.

*3. Have the authors made all data underlying the findings in their manuscript fully available?

Reviewer #1: Yes
Reviewer #2: Yes
Reviewer #3: Yes*

Authors’ Response: Thank you for your positive comments.

*4. Is the manuscript presented in an intelligible fashion and written in standard English?

Reviewer #1: Yes
Reviewer #2: No
Reviewer #3: No*

Authors’ Response: We have revised all the specific errors pointed out by the reviewers. The revised manuscript has also been edited by a native English speaker.

*5. Review Comments to the Author*

Reviewer #1:

*• SUMMARY:*

*The article provides an analysis of differentiating which clinical tests that are best to be performed to diagnose a common binocular vision anomaly, i.e. the convergence insufficiency (CI). The complexity of the anomaly makes practitioners confused and sometimes missed out in detecting and diagnosing CI. As such, this article highlights the important clinical tests and criteria that could help practitioner by performing the Receiver operating characteristic (ROC) curve analysis. While the ROC analysis were well performed, there are a few issues that needs to be addressed as stated below.*

Response 1 to Reviewer #1 (R1R#1): Thank you for these positive comments. We have carefully revised the initial manuscript based on your constructive and helpful comments.

*• FEEDBACK:

1. Materials & Methods Section*

*• Line 99*

*• Please also clarify if participants recruited have not had any vision therapy or eye exercise treatment prior to the enrollment as research participant.*

R2R#1: ‘Participants had not previously undergone any vison therapy or eye exercise treatment.’ was added in lines 104-105 of the revised manuscript.

*• Line 112*

*• The authors should detailed the NPC measurement whether it was done in a free space using accommodative fixation target or using the RAF ruler.*

*• MAJOR: Target used for the test (20/50) was too large for the 20/20 research participants group. Using this target will not exert the best binocular vergence ability of the participants and would affect the whole test outcomes. Please clarify.*

R3R#1: Thank you for this detailed comment. 1) ‘In free space’ and ‘on a Gulden fixation stick’ were added to the methods section. 2) A large target may not be able to achieve the best binocular vergence ability, as the reviewer has pointed out. However, various targets can be used to measure NPC, such as near accommodative targets (different sizes in the range of 20/25 to 20/200), pencil tip, penlight, and penlight with red/green glasses [R1]. Several studies have stated the differences in the NPC with different target types are not likely to be clinically significant [R2], and the detail of the accommodative target is also irrelevant as there are no significant differences between a fingertip, pencil tip, or N5 letter presented in free space [R3]. In addition, it has been suggested that a target with fine detail should be avoided, otherwise patients often confuse blur with diplopia [R4]. Therefore, we chose to use the isolated 20/50 target to measure NPC [R5, R6].

Reference:

[R1] Carlson NB, Kurtz D. Clinical procedures for ocular examination, 3rd ed. New York: The McGraw-Hill; 2004. pp. 50–51.

[R2] Siderov J, Chiu SC, Waugh SJ. Differences in the nearpoint of convergence with target type. Ophthalmic Physiol Opt. 2001; 21(5):356-360.

[R3] Adler PM, Cregg M, Viollier AJ, Margaret Woodhouse J. Influence of target type and RAF rule on the measurement of near point of convergence. Ophthalmic Physiol Opt. 2007; 27(1):22-30.

[R4] Elliott D. Clinical procedures in primary eye care, 3rd ed. Edinburgh: Butterworth-Heinemann; 2007. pp. 188–190.

[R5] Scheiman M. Wick B. Clinical management of binocular vision: heterophoric, accommodative, and eye movement disorders, 4th ed. Philadelphia: Lippincott Williams & Wilkins; 2014. pp. 43–44.

[R6] Ostadimoghaddam H, Hashemi H, Nabovati P, Yekta A, Khabazkhoob M. The distribution of near point of convergence and its association with age, gender and refractive error: a population-based study. Clin Exp Optom. 2017; 100(3):255-259.

*• The authors should specify if the procedures were performed by a sole researcher or a group of researchers. Please also state if the procedures were done in the same order for each participant as some binocular vision assessments are invasive, i.e. will likely introduce fatigue to participants if it is not controlled. This too would affect the whole test outcomes and data collected.*

R4R#1: Thank you for pointing out these details that we had missed. ‘All measurements were taken in a general clinical room by the same examiner, who performed all tests within approximately 30 minutes, using the same methodology.’ were added to lines 106-108.

*2. Results Section*

*• Line 207*

*• Instead of writing the refractive errors and classify them for ‘each eye’, I would suggest that the authors change the way of writing this as per participant, as convergence insufficiency is a binocular anomaly and not a monocular phenomenon.*

R5R#1: We had only presented the characteristics of the participants’ refractive errors in this part. We agree with the reviewer that it would be good to present refractive errors based on both eyes per participant. Therefore, we have added ‘Myopia was present in 125 participants (67.9%), hyperopia in six participants (3.3%), emmetropia in 42 participants (22.8%), mixed anisometropia in four participants (2.2%), simple myopic anisometropia in four participants (2.2%), and simple hyperopic anisometropia in three participants (1.6%).’ to lines 237-240.

*• Line 219*

*• It would be beneficial if the authors add in the normative data (e.g.: Morgan’s Normative Data on Binocular Vision) in Table 2 for the readers to understand the distribution of the data against the normative data used clinically.*

R6R#1: Normative values have been included in the method of each test. References ‘[17, 24]’ related to Morgan's normative data have been included in lines 127, 138, 148, 159, 168, 175, 182, and 188.

*• Line 269*

*• The NPC cut-off written in the paragraph was >7.3cm, different than what is stated in Table 5 (>7.2cm). Please double-check.*

R7R#1: We have rewritten 7.3 cm to 7.2 cm.

*• The sensitivity and specificity for ROC analysis are usually expressed in percentage with 95% confidence interval. The authors may want to revise how they written this to make it more readable for other researchers.*

R8R#1: We think that this choice depends on the authors’ preference or statistics program. Sensitivity is shown on the y axis from 0 to 1 (0–100%), and 1-specificity is shown on the x axis from 0 to 1 (0–100%). The IBM SPSS statistics program used in this study provides a scale of 0–1. The area under curve (AUC) also has a scale of 0–1. Scales of 0–1 are seen frequently in PLoS ONE [R1].
Reference:

[R1] deCastro BR. Cumulative ROC curves for discriminating three or more ordinal

outcomes with cutpoints on a shared continuous measurement scale. PLoS ONE 2019; 14(8): e0221433.

*3. Discussion Section*

*• Although the participants’ groupings and parameters are complicated but the authors managed to write and explain in a good flow. I hope to see a more analytical explanation when discussing the outputs and its relationship to what have been the current knowledge in diagnosing convergence insufficiency. Some comments I have stated above may or may not change the outputs and interpretation of the results.*

R9R#1: Thank you for these detailed and important comments that help us to improve the submitted manuscript. We recognize that the technical expressions are sometimes limited. However, we have done our best to provide a sophisticated explanation and the unclear parts have been revised.

*4. Grammatical errors (see attachment)*

R10R#1: All have been revised according to the reviewer’ comments as follows:

• Line 95 & 97: “,” has replaced by “=” in line 98.

• Line 107 & 108: ‘national university institutional review board’ has replaced by ‘National University Institutional Review Board’; ‘corporation’ has replaced by ‘Corporation’ in lines 102, and 114.

• Line 157: ‘±2.0 D’ has replaced by ‘±2.00 D’ in 177.

• Line 311: ‘vison’ has replaced by ‘vision’ in line 345.

========================

Reviewer #2:

*Generally, the Introduction is well-written. Within the Methods there are missing details which mean that the study could not be reproduced. The Results is the weakest section. It is not clear which participants are included in which groups and the names of the groups were changed throughout the results. Please see the comments below for details. It was difficult to follow the discussion due to the concerns regarding the results. I would recommend making those changes first to allow the reviewer to make more informed comments regarding the discussion.*
R1R#2: Thank you for your positive comments regarding the Introduction. The weakest sections (Methods and Results) have been revised according to the reviewer’s detailed comments, and we have also rechecked the discussion. These comments are very constructive and valuable, and they have helped us to improve the clarity of the manuscript, specifically in terms of our methodology and the goal of this manuscript.

*Introduction
44 – delete ‘common’. There is no evidence provided and later on the prevalence of CI is covered with references.*
R2R#2: We have deleted ‘common’ from the introduction and from the abstract.

*46-8 – no references for the signs of CI*
R3R#2: The signs of CI are well known. However, we have included a reference [1] for the readers to understand the signs for CI.

*65-67 – study 11 not clearly presented. Did their participants have CI? Were they symptomatic? Was ROC curve analysis used? It is not clear how this study is relevant.*
R4R#2: Considering that CI is a nonstrabismic binocular anomaly associated with a large near exophoria, study [11] aimed to analyze the diagnostic validity of accommodative and binocular tests in a sample of patients with a large near exophoria with moderate to severe symptoms, and to analyze their diagnostic validity by means of ROC analysis, sensitivity, specificity, and likelihood ratios. Therefore, ‘A previous study used ROC analysis to investigate the diagnostic validity of the clinical signs of CI in participants aged 19 to 35 years with either symptomatic large exophoria and normal heterophoria, or low visual discomfort at near [11]; it reported that NPC and BAF tests showed the best diagnostic accuracy for discriminating between the two groups’ has been included in lines 65-69.

*69 – did study 12 test NPC? This was the test that showed best diagnostic ability in the other studies. I would assume this would be tested in this study investigating CI.*

R5R#2: Study [12] focused on only BAF and VF. In this study, participants were classified into the CI and normal groups using the Convergence Insufficiency Symptom Survey (CISS). These descriptions have been included in lines 67-69.

*77-84 This is an unclear p/g. The first approach requires the analysis of signs measured by each test, as does the second approach. It is not clear what the difference is. Do you mean the final ‘approach’ rather than ‘stage’? The 3rd approach also needs to be explained more clearly.*

R6R#2: Thank you for these detailed comments. The first approach is used to distinguish CI from accommodative dysfunction. We have replaced ‘stage’ with ‘approach’. As the 3rd approach relates to the lines 80-84, we linked the first and last sentences.

86 – *it is not clear why fusional vergence has been explicitly stated, whilst the others are all put under ‘signs’.*
R7R#2: In vergence disorders such as CI, the measurement of fusional vergence is an important in evaluating ability to maintain single binocular vision. Therefore, in CI screening, fusional vergence should be evaluated due to one of signs related to CI. We have added ‘related to ability to maintain single binocular vision’ to line 90.

*Methods

In the introduction it was stated that the students had ‘visited for primary eye care’. That should appear in the method section instead and it should be stated whether this was in a hospital setting. It is not clear how they were recruited. Was the project advertised to seek students with ocular discomfort? Or were University students identified within the primary eye care and asked to partake?

How was it determined that these students had ocular discomfort? Were they asked any questions regarding this?*

R8R#2: Thank you for pointing out these details that we missed. ‘Participating students voluntarily visited a university eye clinic center for primary eye care due to blurred vision, eyestrain, and visual discomfort.’ was added to lines 96-101. History taking related to ocular symptoms has been performed to find out the purpose of student’s visit as general process for eye examination.

*96 - Were set questions asked about the ocular discomfort?*

R9R#2: The Suggested Questions for Patient History described in reference [1] were followed. The reference was cited in line 107.

*105 – replace ‘by an ocular motility and’ with ‘by ocular motility testing and’*

R10R#2: We have revised the text according to the reviewer’s comment.

*107 - It is not clear how the phoropter was used for binocular vision tests*
R11R#2: The phoropter is an instrument invented in the early 1900s and used to measure the ametropias, phoria, and the amplitude of accommodation of the eyes. The power of lenses in the phoropter can be modified in steps of 0.25 D. It is commonly used by eye care professionals during an eye examination. We think that a description of how to use it is not necessary.

*108 – tests were performed at distance ‘or’ near – should this be ‘and’?*

R12R#2: We have revised the text according to the reviewer’s comment.

*112-6 – was the distance for NPC recorded in cm? How was the speed of the target controlled to 5cm/s? ‘at the eye level and between the both eyes’ should be ‘between both eyes and at eye level’.*
R13R#2: ‘(cm)’ in line 124, ‘approximately’ in line 126, and ‘between both eyes and at eye level’ in line 127 were included or revised. The speed of the target was approximated considering the rate of the patient’s reaction.

*122 – how was the speed controlled to 2PD per sec?
When measuring the accommodative amplitude, how was the speed controlled? What were the instructions? Were they told to report when the target first became blurred or when it was so blurred they could read the letters? The last word ‘accommodation’ should state ‘(in meters).*

R14R#2: Speeds that were either too slow or too fast could affect the measured values, so we tried to regulate the speed for measurement to rule out this affect. ‘the subject reported when the target first became blurred while moving’ in lines 145-149, ‘(cm)’ ‘approximately’ in line 146, and ‘(m)’ in line 148 were included in the revised text.

*139 – this reference no. 18 seems inappropriate. Is it to justify the method being used? The use of references need to be considered as no. 17 (129) also appeared inappropriate.*
R15R#2: References [17] and [18] were replaced with [25] (renumbered as [17]). The page of reference [25] was changed from pp. 52-76 to pp. 36-76.

*142 – the word ‘prism’ is missing*

R16R#2: We have revised the text accordingly.

*143 – Relating back to my point re. 139, it would be more appropriate to have a reference here to state why testing was done at near first.*

R17R#2: Reference [17] was cited in line 153.

*The word AC/A should always be followed by ‘ratio’. Why was 1D used? It is typically measured using 3D. Either explain or use a reference to justify the method. The distance was divided by 2.5D, presumably because the test was conducted at 40cm, but this should be stated somewhere. There was also not enough detail on how the lenses were introduced and how IPD was measured? Was this all done using the phoropter?*

R18R#2: Thank you for your detailed comments. AC/A means the ratio of AC to A. Therefore, we think that ‘ratio’ is not necessary. We have defined ‘Gradient AC/A’ as ‘Gradient AC/A ratio (AC/A)’ in line 164. Gradient AC/A can be determined by adding +3D to -3D. We used +1.00D considering the addition of plus power (less minus power) in a binocular vision prescription. In a phoria prescription, the preference of the prescription is in order of vision training (VT), addition of plus power (as the same maximum plus power for maximum visual acuity), prism prescription, and so on. IPD was measured with a PD meter. Reference [17] was included. Lines 166–167 were partly revised.

*153 – Was the phoropter used to change lens strengths? Normative values should be presented here, and for all the other tests used.*
R19R#2: In the ‘General procedure’ section, the use of the phoropter has been stated. As mentioned in the response of R11R#2, the lens power in the phoropter can be changed by steps of 0.25 D. We have provided normative values, as requested.

*Was BAF tested at 40cm? Normative values from the literature would also be helpful.*
R20R#2: BAF was tested at 40cm. 40cm was included in line 177. Normative values were presented from thein literature [17, 24].

*Vergence facility testing is the main concern of the method section. The method described does not test VF. This would require a BI and a BO prism, yet the current study used a BI and BU prism. Is this a typing error? The subject should also be reporting when the target becomes single, not when they note clear vision.*
R21R#2: Thank you for these valuable comments. This is a typing error. ‘clear vision’ has been replaced with ‘single vision’.

*The method used to calculate Percival’s criterion is not clearly presented.*

R22R#2: Percival’s criterion was stated in line 194-197. In these lines, prism = (1/3) (greater of the two fusional vergence) – (2/3) (lesser of the two fusional vergence) was described.

*179-83 – What is Morgan’s? Is this a reference? It is not in the reference list either. So were all participants used and categorised into one of the 10 diagnoses? It is not clear what the expected criteria is for each test? Do you mean the expected normative values? These could have been presented when describing each of the tests. It is not clear what modification were made from the study by Scheiman and Wick.*

R23R#2: Morgan's refers to Morgan’s table of expected findings for each test. Morgan’s expected values for each test were included in references [17, 24]. Normative values of each test have been included in the methods section. This ‘which were modified from’ is a describing error. ‘which were modified from’ have been replaced with ‘which referred to’.

*Table 1 – ‘1 and 2 or 3’…does this mean you need both signs 1 and 2, or just sign 3? Or does this mean you need sign 1 as well as sign 2 or 3? If it is the first option, then perhaps ‘signs 1 and 2, or sign 3’ would be better? If it is the second option, perhaps put this as ‘presence of sign 1 and sign 2 or 3’.*

R24R#2: Table 1 has revised as ‘sign 1 and sign 2 or 3’.

*Table 1 – What is meant by a ‘normal phoria’? What counts as ‘reduced’ PFV and NFV? Can abbreviate binocular accommodative facility under AI. Should BAF be more than 13 cpm in AE? Is a reference required for this table if it has been taken and modified from another source?*

R25R#2: We have included additional explanations to improve the clarity of the table. References have been cited as stated in R23R#2. BAF should be less than 13 cpm in AE due to fail for +2.00D.

*194-204 – Which 3 groups were compared? It has not been stated that the subjects were put into 3 groups. I had assumed subjects were put into the 10 diagnostic categories based on the information that has been provided. Should the sentence on specificity and sensitivity should come before it was stated that the ROC was calculated because the sensitivity values are used for the ROC? It would be useful to state this and 1-specificity are used to plot the curve. Perhaps I am mistaken but I think the ‘cut-off’ is the value that you use to decide what test result indicates whether or not someone has CI, and therefore would affect the sensitivity and specificity, and therefore would affect the curve. How was the AUC calculated? It would be useful to indicate that a AUC value of 1 and 0.5 are important and why.*

R26R#2: The three groups were all CI, binocular vision anomaly except CI (BVA), and normal binocular vision (NBV). The groups are described in lines 202-203. ‘by plotting sensitivity on the y axis as a function of 1 – specificity on the x axis’ have added to line 220. ‘The cut-off was also identified for each test with the largest AUC which was significantly greater than 0.50.’ has been added to lines 228-229.

Results

*Were all the subjects with a refractive error wearing their refractive correction for testing?*

R27R#2: ‘after wearing refractive correction’ has been added to lines 241-242 to clarify this.

*211 – Here it is stated what the 3 groups are but this should have appeared in the method section. The groups are unclearly presented. What is meant by ‘all CI’? Is this anyone that fell into the CI category in Table 1? Are those in ‘BVA’ anyone that falls into any other diagnosis based on Table 1? I assume NBV are those that have nothing wrong and it might be less confusing to refer to them as the control group. The abbreviation BVA is also confusing as that typically stands for binocular visual acuity*

R28R#2: In relation to the answer given in R26R#2, a description of the three groups has been noted to lines 203-208 of the method section as follows: ‘Subjects groups were classified into three groups according to Table 1: All CI including CI with 2 signs and CI with an accommodative dysfunction, other binocular vision anomaly (BVA) except CI, and normal binocular vision (NBV). All CI were also classified into CI with 3 signs (CI3), CI with 2 signs and an accommodative dysfunction (CI3AD), and CI with 2 signs (CI2).’ The abbreviation BVA was determined under our careful consideration.

*Table 2 – The AA for All CI is reported to be 1.00D – is this correct? The significance results do not match up to those reported in the text on page 10. E.g. AA was reported to be significant on 214 but here it is not significant. NPC is reported to be significant in the table but is not reported on page 10. Phoria at distance in the table states that c is significant greater than b, but on page 10 it states that there was a significant difference between all CI and other BVA. These all need to be checked.*
R29R#2: Thank you for this detailed check of the data. 10.90 D is the correct AA for all CI; we have revised the table 2 to reflect this. We have deleted AA and added NPC in line 245. There was a significant difference between all CI and other BVA, and other BVA and NBV in distance phoria and distance NFV. The text has been revised to make this clear.

*229 – ‘non-strabismic binocular vision anomalies’ – does this refer to everyone in all CI and other BVA but excluding those in NBV? It is confusing as the terms keep interchanging. On line 232 is the NBV group now referred to as the ‘normal group’?*

R30R#2: ‘non-strabismic binocular vision anomalies’ means all CI and other BVA except NBV. ‘non-strabismic’ was deleted to eliminate confusion in line 261 and from the title of Table 3. "normal group" has been replaced with "NBV" to avoid confusion in line 264.

*232-38 Within this p/g indicate CI2AD etc so that it matches with the terms used in Table 3.*
R31R#2: The paragraph has been revised to use NBV, CI2, CI + AI, CI + AE, CI3, and CI2AD so that it matches with the terms used in Table 3.

Table 3 – Again it is unclear who fits within the various groups. Does the ‘non-CI’ group contain everyone in the ‘other BVA’ group? Looking at the numbers it looks like this could be those in ‘other BVA’ and ‘NBV’. Does AD include anyone of those listed with AE and AI? I would assume so but the numbers don’t add up correctly. Why is FVD and accommodative infacility not included in this table?

R32R#2: ‘Non-CI corresponding to CI is represented in the non-CI group.’ has been included in Table 3 to improve its clarity. All (including NBV) except classified CI (CI3, CI3AD, CI2, CI2AD) have been classified into the non-CI group. In the case of this study, FVD and accommodative infacility was not present.

*246-53 – ‘for each CI test’ is unclear. Firstly, it would be better to just refer to them as ‘tests’ rather than ‘CI tests’ and secondly, they are not all included in Table 4. Again it is stated ‘for each test’ yet Figure 1 only shows the ROC curves for NPC, Sheard and Percival. State why only particular tests are presented in Table 4 and figure 1. I assume ‘for diagnostic tests excluded in Table 1’ refers to Sheard and Percival? This should be explicitly stated, rather than requiring the reader to go back and work this out.*

R33R#2: ‘ROC curve analysis was performed for the tests shown in Table 2. The results of the AUC for NPC, Sheard’s and Percival’s criterion with p < 0.05 and AUC > 0.5 in 95% confidence interval ranges, and PFV, NFV, and AC/A including the diagnostic criteria in Table 1 are shown in Table 4’ has been added to line 278-281. ‘Fig 1 shows ROC curves for NPC, Sheard’s and Percival’s criterion’ have been included in 281-282. The reason for ‘why only particular tests are presented in Table 4 and figure 1’ is that other tests except tests presented in Table 4 and Figure 1 were not significant and tests resented in Table 4 and Figure 1 were significant and high priority for CI screening (Figure R1).

| For NPC, Sheard’s, and Percival’s criterion  (Fig 1) | For all tests |
| --- | --- |
|  |    A. CI3 + NBV |
|  |   H. CI2AD + non-CI |

Figure R1. Example: comparison of ROC curves for particular tests (Fig 1) and all tests.

*253 – ‘AUCs were greater for NBV than for non-CI’. In an earlier comment I had stated that it seemed that ‘non-CI’ included those in ‘other BVA’ and ‘NBV’, yet now it is suggested that this is not the case.*

R34R#2: As in R30R#2 and R32R#2, when CI is classified as CI3, CI3AD, CI2, and CI2AD, respectively, the non-CI group includes all (other BVA and NBV) those except for the classified CI, respectively. For an example, Table 4 represents the ability to discriminate CI from CI3 + NBV, and discriminate CI from CI3 + non-CI.

*Table 4 – How was it chosen which tests to present here? For NFV, how was it chosen whether to present break or recovery point and near or distance result? It is not clear what is meant by each of the ‘screening for various CI conditions’, e.g. are you comparing the groups, so CI3 versus NBV? Etc. The paragraph from 267 suggests you are combining the stated groups but it is not clear why you would be doing this.*

R35R#2: We chose the tests to present here based on 1) Fig 1 and tests having p < 0.05 and AUC > 0.5 from all tests in Table 2, and 2) PFV, NFV, and AC/A including diagnostic criteria in Table 1. NFV for distance has been represented in a footnote in Table 4. ‘screening for various CI conditions’ has been revised to 'Ability to discriminate CI" to improve the clarity of Table 4. To further reduce confusion, the first column of Table 4 has also been revised.

*Figure 1 – It is not clear which is the dotted and dashed line. Perhaps a coloured figure would be more appropriate.*

R36R#2: Figures have been revised and are now represented by colors.

*301 – should state ‘was also evaluated in NBV and non-CI conditions’?*
R37R#2: We have corrected the text to ‘was also evaluated in NBV and non-CI conditions’.

*308 – the accommodative conditions were abbreviated earlier. Also, what about accommodative infacility?*
R38R#2: ‘accommodative insufficient (AI)’ has been changed to ‘AI’, ‘accommodative infacility’ had not been abbreviated.

*311-2 – This sentence does not seem to link with the previous sentence.*
R39R#2: This sentence has been corrected to ‘CI should also be distinguished from conditions such as normal vision, diverse vergence and accommodative disorders, and other binocular anomalies.’

*327 – unclear why discriminating CI from abnormal rather than normal groups.*

R40R#2: ‘from abnormal groups combined with other binocular anomalies’ has been added to lines 361-356 to clarify this.

========================

Reviewer #3:

*This study was aimed to evaluate the ability of screening tests to discriminate convergence insufficiency, that is very important issue in the fields of heath science and related areas. The authors are presenting interesting results, however the manuscript should be improved:*

R1R#3: Thank you for these positive comments. We have revised the manuscript according to your comments.

*1. More information about criteria to select the participants should be added in the materials method section.*

R2R#3: We have rechecked the manuscript, and have revised the unclear parts of the text. To summarize the inclusion criteria of participants, participants were university students, voluntarily visited with ocular discomfort, had no previous vision therapy or eye exercise treatment, no ocular disease and surgery, and did not have amblyopia and strabismus. These criteria have been added to line 95-101.

*2. Have research group got the informed consents from study subjects, that information should be clearly indicated in the paper*

R3R#3: We have clarified this by adding ‘with waiver of the informed consent for the retrospective collection of clinical data’ to lines 103-104.

*3. Why did author consider the significantly difference when A p-value of ≤ 0.05, is that should be A p-value of < 0.05?*

R4R#3: We have replaced ‘A p-value of ≤ 0.05’ with ‘A p-value of < 0.05’.

*4. The Table 1: is the criteria made by the authors, or from others? If from others these should be some citations*

R5R#3: The criteria of Table 1 were obtained from Scheiman and Wick’s study [17] and the expected criteria for each test [24]. This is explained in lines 181-183 in the submitted manuscript (208-210 in the revised manuscript).

Reference:

[17] Scheiman M. Wick B. Clinical management of binocular vision: heterophoric, accommodative, and eye movement disorders, 4th ed. Philadelphia: Lippincott Williams & Wilkins; 2014. pp. 36-76.

[24] Benjamin WJ. Borish’s clinical refraction, 2nd ed. St. Louis: Butterworth-Heinemann; 2006. pp. 968-972.

*5. Why the figure 1 does not have the legend?*

R6R#3: The legend for Figure 1 can be found in lines 296-299. The legend has been placed in the text near to the first mention of Figure 1, as per the PLOS ONE guidelines.

*6. English errors and writing style should be checked by native English speakers*

R7R#3: Our manuscript has been checked by Editage for native English language editing.

- The end of response to reviewers’ comments –
