# Supplementary material for: Receiver operating characteristic curve analysis of clinical signs for screening of convergence insufficiency in young adults
Source: PLoS One. 2020 Jan 24;15(1):e0228313. doi: 10.1371/journal.pone.0228313 (PMC6980576; doi:10.1371/journal.pone.0228313)
Supplement: Attachment — Submitted filename: 2nd Response_to_Reviewers.docx [file pone.0228313.s005.docx]

Comments to the author:

1. If the authors have adequately addressed your comments raised in a previous round of review and you feel that this manuscript is now acceptable for publication, you may indicate that here to bypass the “Comments to the Author” section, enter your conflict of interest statement in the “Confidential to Editor” section, and submit your "Accept" recommendation.
Reviewer #1: All comments have been addressed
Reviewer #2: All comments have been addressed
Reviewer #3: (No Response)

**Authors’ Response**: We thank the reviewers their positive comments.

2. Is the manuscript technically sound, and do the data support the conclusions?
Reviewer #1: Partly
Reviewer #2: Yes
Reviewer #3: (No Response)

**Authors’ Response**: We have revised the manuscript in view of Reviewer #1’s comments. We have stated the revisions we have made in the R1R#1.

3. Has the statistical analysis been performed appropriately and rigorously?
Reviewer #1: Yes
Reviewer #2: Yes
Reviewer #3: Yes

**Authors’ Response**: We thank the reviewers for their positive comment.

4. Have the authors made all data underlying the findings in their manuscript fully available?
Reviewer #1: Yes
Reviewer #2: Yes
Reviewer #3: Yes

**Authors’ Response**: Thank you for these positive comments.

5. Is the manuscript presented in an intelligible fashion and written in standard English?
Reviewer #1: No
Reviewer #2: Yes
Reviewer #3: Yes

**Authors’ Response**: Our manuscript has been revised in view of Reviewer #1’s comments (the attached file–[PONE-D-19-17984_R1_(2nd Review).pdf](https://www.editorialmanager.com/pone/download.aspx?guid=%7b2fdc33d9-f0f8-4cfe-a388-422310afc8cb%7d&scheme=13&id=391469)).

We have given more details of the revisions we have stated in the R2R#1– R10R#1.

6. Review Comments to the Author

(Reviewer’s comments are shown in *Italic*)

Reviewer #1:

*Need clarification on data distribution.*
**Authors’ Response (R1R#1)**: We think that the statement ‘Need clarification on data distribution’ is referring to the reviewer’s comment “One-way ANOVA with a Bonferroni’s post-hoc test– Were the analyses performed under the assumption that the data were normally distributed? Please clarify and state here.” in line 217 in the Data analysis section. In response to this comment, we have made the following revisions to our manuscript:

1) We have added the following text to the manuscript: ‘The descriptive statistical analysis was based on mean and standard deviation values. Prior to ANOVA analysis, the data was checked for normality using the Kolmogorov–Smirnov test. A Kruskal–Wallis one-way ANOVA (nonparametric test) with a Dunn–Bonferroni’s *post-hoc* test comparing the three groups (all CI, BVA, and NBV) was performed’ in lines 218–221.
2) We have also partly revised in the Table 2 and lines 247–248.

*The manuscript still needs tidying up in terms of language and style (please refer to the highlighted comments in the attchment).*

**R2R#1**: Our revised manuscript has also been rechecked by a native English-speaker.

*Line 68: The phrase ‘near [11]; it reported’ is too long. Should be a 'full stop' here.*

**R3R#1**: We have added a full stop to split this long sentence into two in line 68.

*Line 92: Take this ‘for’ in ‘who visited for primary eye care’ out!*

**R4R#1**: We have deleted ‘for’ in line 93.

*Line 93: CI screening or screening of CI in ‘screening CI’.*

**R5R#1**: We have changed ‘screening CI’ to ‘CI screening’ in line 94.

*Line 126: ‘rate of approximately 5 cm/s’, I personally think that this should be taken out as it is a bit controversial unless the authors have a proper technique(s) to measure this that can be replicated*.

**R6R#1**: We have changed ‘rate of approximately 5 cm/s’ to ‘slowly’ in line 127.

*Line 134: ‘a rate of approximately 2 Δ/s’, I personally think that this should be taken out as it is a bit controversial unless the authors have a proper technique(s) to measure this that can be replicated.*

**R7R#1**: We have changed ‘a rate of approximately 2 Δ/s’ to ‘slowly’ in line 135.

*Line 217: ‘One-way ANOVA with a Bonferroni’s post-hoc test’, Were the analyses performed under the assumption that the data were normally distributed? Please clarify and state here.*

**R8R#1**: We have revised the text as follows: ‘The descriptive statistical analysis was based on mean and standard deviation values. Prior to ANOVA analysis, the data was checked for normality using the Kolmogorov–Smirnov test. A Kruskal–Wallis one-way ANOVA (nonparametric test) with a Dunn–Bonferroni’s post-hoc test comparing the three groups (all CI, BVA, and NBV) was performed’ in line 218–221.

*Line 382: take this ‘that’ out.*

**R9R#1**: We have deleted ‘that’ in line 386.

*Line 456: ‘were’*

**R10R#1**: We have changed ‘were’ to ‘are’ in line 460.

------------------------
Reviewer #2:

*Thank you for addressing my comments and feedback. The paper is much clearer. The method and results section are much easier to follow. I have no further comments.*

**R1R#2**: Thank you very much for this kind review.

------------------------
Reviewer #3:

*Thank for addressing my comments! I think you authors have worked hard to revise the manuscript as required by the reviewers*

**R1R#3**: Thank you for your positive comments.

- The end of response to reviewers’ comments –
